# An integrated study of Violae Herba (*Viola philippica*) and five adulterants by morphology, chemical compositions and chloroplast genomes: insights into its certified plant origin

**DOI:** 10.1186/s13020-022-00585-9

**Published:** 2022-03-03

**Authors:** Gengyu Lu, Juanjuan Qiao, Long Wang, Hui Liu, Gang Wu, Yan Zhu, Yucheng Zhao, Guoyong Xie, Minjian Qin

**Affiliations:** 1 Department of Resources Science of Traditional Chinese Medicines, School of Traditional Chinese Pharmacy, China Pharmaceutical University, Nanjing, 211198 China; 2 State Key Laboratory of Natural Medicines, School of Traditional Chinese Pharmacy, China Pharmaceutical University, Nanjing, 210009 China; 3 Yangzhou Center for Food and Drug Control, Yangzhou, 225000 China

**Keywords:** Integrated study, Violae Herba, *Viola*, Herbgenomics, Chloroplast genome, Superbarcodes, Pharmacopoeia revision

## Abstract

**Background:**

*Viola philippica* Cav. is the only original plant for Violae Herba, as described in the Chinese Pharmacopoeia. The quality of this crude drug is affected by several adulterants from congeneric *Viola* species, and the authentic plant origin of Violae Herba is still controversial. Genome-based identification offers abundant genetic information and potential molecular markers that can be used for the authentication of closely related species. This study aims to investigate the certified origin of Violae Herba and to develop more effective markers for these easily confused species at the genetic level.

**Methods:**

We compared the morphology and chemical composition of 18 batches of commercial samples and six widespread medicinal *Viola* plants used as Violae Herba or its substitutes by TLC and HPLC-Triple-TOF–MS/MS analyses. The complete chloroplast genomes of these species were sequenced and analyzed, including the general features, repeat sequences, mutational hotspots and phylogeny. The complete chloroplast genomes used as superbarcodes and some specific barcodes screened from mutational hotspots were tested for their ability to distinguish *Viola* species.

**Results:**

A comparative study showed that Violae Herba is a multi-origin traditional Chinese medicine. Commercial decoction pieces and the standard reference drug were mainly derived from *V. prionantha*, clashing with the record in the Chinese Pharmacopoeia. Chloroplast genome analyses of *V. philippica* and five adulterants indicated that sequence divergence was relatively low within *Viola* species. By tree-based approaches, the complete chloroplast genomes showed a better discrimination ability and phylogenetic resolution for each *Viola* species. These results indicate that the whole chloroplast genomes can be used as superbarcodes to differentiate *Viola* medicinal plants. More specific DNA barcodes could be further developed from the *Viola* chloroplast genomes for more efficient and rapid identification of commercial Violae Herba and its adulterants.

**Conclusions:**

This study has implications for chloroplast genome-based phylogenetic analysis and the authentication of multiple *Viola* species used as Violae Herba. The legal origin recorded in the Chinese Pharmacopoeia should be further revised to *V. prionantha*, in line with the commercial Violae Herba in the TCM markets.

**Supplementary Information:**

The online version contains supplementary material available at 10.1186/s13020-022-00585-9.

## Background

Violae Herba (VH), also called “Zi Hua Di Ding” or “Di Ding” in Chinese, is one of the most common traditional Chinese medicines [1]. However, from illustrations in the historical literature, the plant origin of “Zi Hua Di Ding” has always been ambivalent. Since ancient times, different species from Fabaceae, Papaveraceae, Polygalaceae, and Violaceae have been described as “Zi Hua Di Ding” [2]. *Viola yedoensis* Makino was specified as the unique origin of “Zi Hua Di Ding” (Violae Herba) in the Chinese Pharmacopoeia (ChP) since the 1977 edition and is now treated as a synonym of *V. philippica* Cav. [3, 4]. Currently, “Zi Hua Di Ding” in traditional Chinese medicine (TCM) markets mainly refers to the whole plant of species in the genus *Viola*. VH is traditionally used as a heat-clearing and toxin-resolving medicine in TCM prescriptions to treat hepatitis and other infections [5]. As the vegetative period of *Viola* varies, it is extremely difficult to ensure source uniqueness. The effective components and pharmacological effects are significantly different between VH and its adulterants, and there has been considerable confusion in its identification and application [6–9]. Morphological, pharmacognostic and chemical methods have been applied to identify members of this phytogroup [10–12]. Although there are numerous studies of VH and its sources, controversy still exists with respect to its authentic source. By TCM market investigation, we learned that VH is often adulterated by other similar *Viola* species, and the legal origin *V. philippica* could hardly meet the ChP standard. Thus, we suspect that the certified plant origin in the ChP may be misrecorded.

In recent years, due to the rapid advances in DNA identification using ITS, ISSR, RAPD, and chloroplast (cp) markers [13, 14], molecular tools have been gradually applied in *Viola* research. The nuclear ITS gene and several cp genomic regions (including *trnL-trnF*, *psbA-trnH*, and *rpl16*) have been frequently used in molecular systematics and species authentication of *Viola* [15, 16]. However, the existence of natural hybridization, cleistogamy, and polyploidy in *Viola* makes it difficult to classify [17, 18]. Taxonomy and phylogenetic analyses of *Viola* based on nuclear gene segments and plastids led to conclusions that were inconsistent with traditional views. Thus, common single-locus markers have failed to provide sufficient genetic variation information to elucidate the infrageneric relationships among *Viola* species; therefore, more reliable barcodes are urgently needed.

The development of omics-based analysis on herbal medicines has led us to enter the “herbgenomics” era [19]. Genomic information is an important tool for investigating evolution and divergence and is useful for clarifying the morphological variation as well as the phylogeny of specific phytogroups [20]. However, only a few whole genomes are available, given the complexity of their assembly [21]. Chloroplasts are considered semiautonomous organelles that have relatively independent genetic material [22]. Compared with those of nuclear genes, the genome structure, gene content, and gene order of cp genes are more conserved [23], which has great significance in plant phylogenetic analysis and species identification when using whole cp genomes as “superbarcodes” [24–27].

Next-generation sequencing (NGS) has made obtaining cp genomes less expensive and more feasible. In the case of the genus *Viola*, the complete cp genomes of several Korean species have been published and analyzed [28, 29]. In this study, we compared the morphological and chemical composition of 18 batches of commercial VH with the original plant *V. philippica* and five other widespread common adulterants: *V. inconspicua* Blume, *V. betonicifolia* Sm., *V. japonica* Langsd. ex Ging., *V. collina* Besser, and *V. prionantha* Bunge. We then performed comparative analyses of the complete cp genomes of these six species. The main purpose of this study is to clarify the certified origin of VH and to develop more effective markers and superbarcodes for these easily confused species at the genetic level.

## Methods

### TCM market research and qualitative analysis

Eighteen batches of commercial VH samples purchased from nine TCM markets from Anhui, Hebei, Henan, Hubei, Jiangsu, Shandong, Shaanxi, Sichuan, and Guangdong provinces were examined (Additional file 1: Table S1). The dried leaves of each sample were gently expanded in water. Whole herbs identified as substitutes and adulterants of VH were collected in different provinces of China and photographed (Additional file 2: Table S2). All samples were identified by Professor Minjian Qin (China Pharmaceutical University).

Thin-layer chromatography (TLC) was conducted according to the standard methods of the ChP [30]. Chromatograms were inspected using a TLC scanner. The reference substance (esculetin) and standard reference drug (Violae Herba No. 121429-201605) were purchased from the National Institute for Food and Drug Control (NIFDC).

### Chemical composition analysis

High-performance liquid chromatography coupled with diode array detection (HPLC–DAD, Agilent Series 1260) was used to analyze the chemical compositions of these samples. The dry powders of all the dried samples and the reference drug (1.0 g; 60 mesh) were sonicated in 80% methanol (15 mL) at room temperature for 30 min, and the extracts were centrifuged at 10,000 rpm for 15 min. The supernatant was transferred to a 25 mL brown volumetric flask and diluted to volume with 80% methanol. The samples were filtered through a 0.22 μm microfiltration membrane before HPLC. The main ingredients of the six *Viola* species were determined by high-performance liquid chromatography coupled with triple time-of-flight mass spectrometry (HPLC-Triple-TOF–MS/MS, AB SCIEX TripleTOF 4600).

Chromatography was performed at 25 °C on an Agilent ZORBAX SB-C18 (4.6 × 250 mm, I.D. 5 μm) with a flow rate of 1 mL/min; the injection volume was 20 µL. The mobile phase consisted of solvents A (0.4% aqueous acetic acid, v/v) and B (methanol). The gradient elution program was as follows: 0–10 min, 5–25% B; 10–18 min, 25–30% B; 18–30 min, 30–35% B; 30–50 min, 35–45% B; and 50–60 min, 45–95% B. The DAD was set to 345 nm.

The MS/MS analysis was performed in negative and positive ion mode with the following parameter settings: survey scan of 100–1500 Da (250 ms accumulation time) and MS/MS survey scan of 100–1500 Da (100 ms accumulation time) with a declustering potential of 100 V; an ion source voltage of − 4500 V for negative mode and 5500 V for positive mode; an ion source heater at 550 °C; ion source gas 1 at 50 psi; ion source gas 2 at 50 psi; and a collision energy of 44 V.

Characteristic chromatograms for each plant sample were generated by the Similarity Evaluation System for the Chromatographic Fingerprint of TCM (Version 2012.130723) and analyzed by OriginPro (Version 2019b, OriginLab Corporation).

### Genetic analysis of collected and commercial VH samples

The nuclear ITS gene is inefficient for delimiting *Viola* species [15, 31]. Portions of the *rbcL* gene and *psbA–trnH* spacer from the cp genome have been recommended as a two-locus universal barcode for species discrimination [32]. The modified CTAB method was used to extract total genomic DNA from dried samples [33]. The sequence was amplified using the universal primers for *rbcL*a and *psbA-trnH* and previously published PCR conditions [34]. Amplification products were examined by electrophoresis in 1.5% (wt/vol) agarose gels. Qualified PCR products were submitted to GENEWIZ (Suzhou Biotechnology Co., Ltd.) for bidirectional sequencing. Sequences were edited and assembled using BioEdit (version 7.2.5) and manually curated [35]. All *rbcL*a and *psbA-trnH* sequences were uploaded to GenBank.

### Plant materials for cp genome sequencing and DNA extraction

Fresh leaves for cp genome sequencing were collected from grassy areas in Jiaxing, Nanjing, and Shijiazhuang City, China (Additional file 3: Table S3). Voucher specimens were deposited at the Center of Herbarium, China Pharmaceutical University, Nanjing, China (Herbarium Code: CPU). Whole genomic DNA was extracted from fresh leaves using a rapid plant genomic DNA isolation kit (Sangon Biotech Co., Ltd, Shanghai, China). The quality of DNA was checked using a BioPhotometer Plus (Nucleic acid protein detector, Eppendorf, Germany) and 1% agarose gels. Finally, high-quality DNA was sent to GENEWIZ (Suzhou Biotechnology Co., Ltd.) for sequencing.

### Illumina sequencing, assembly and annotation

Sequencing was performed using an Illumina HiSeq X Ten system (GENEWIZ Suzhou, China). The *V. websteri* chloroplast genome was used as a reference (GenBank Accession No. MH229819). Data were assembled using Velvet v1.2.10 and NOVOPlasty v2.7.2 [36, 37]. Complete cp genomes were annotated and analyzed using the online tool GeSeq [38]. The final annotated cp genome sequences were submitted and deposited in GenBank under Bioproject No. PRJNA636230. The circular genome map was drawn using the OGDRAW program based on the annotated results [39].

### Codon usage bias and RNA editing site prediction

CodonW 1.4.2, CUSP and CHIPS of EMBOSS were used to analyze the codon usage, GC3 content, and effective number of codons (ENC) of the protein-coding genes [40, 41]. Putative RNA editing sites in 35 protein-coding genes from cp genomes were identified using the PREP (Predictive RNA Editor for Plants) suite of tools [42].

### Repeat analyses

The microsatellite identification tool MISA (https://webblast.ipk-gatersleben.de/misa/) was used to detect simple sequence repeats (SSRs, parameters: minimum repeat numbers were set to 10, 5, 4, 3, 3, and 3 for mono-, di-, tri-, tetra-, penta-, and hexanucleotide repeats, respectively; repeats were more than 10 bp in length) [43]. The maximum length of the sequence between two SSRs to create a compound SSR was 100 bp. REPuter was used to identify dispersed repeats (forward, complement, reverse, and palindromic repeats) [44]. The parameters for REPuter were a minimal repeat size of 30, a Hamming distance of 3, and over 90% identity. Tandem repeats were identified by Tandem Repeats Finder 4.09 (TRF) with default parameters [45]. Each specific region in the genome was designated as one of these repeat types; tandem repeats were classified prior to dispersed repeats if one repeat motif could be identified as both tandem and dispersed repeats.

### Genome comparison and DNA barcode development

The online program IRscope (https://irscope.shinyapps.io/irapp/) was used to identify contraction and expansion at the borders of IR regions [46]. The cp genomes of six *Viola* species were compared using mVISTA software by the Shuffle-LAGAN alignment program with *V. inconspicua* as the reference [47, 48]. Nucleotide diversity (Pi), polymorphic sites, and parsimony informative sites (PICs) were calculated using DnaSP 6.12.03 software by sliding-window analysis (window length: 800 bp, step size: 200 bp) [49].

All datasets were processed using PhyloSuite [50]. Sequences were aligned using MAFFT version 7 [51]. Gap sites were removed with trimAl using the “-strictplus” option [52]. The discrimination ability of specific barcodes and superbarcodes was inferred by the tree-based method using the neighbor-joining algorithm in MEGA X with 2,000 bootstrap replicates [53, 54]. Genetic mean distance analyses were conducted using MEGA X with the K2P model [55].

### Phylogenetic analysis

Phylogenies were constructed using the complete cp genome sequences of 17 *Viola* species, with *Passiflora edulis* as the outgroup. The ModelFinder program was used to select the best-fit model for the aligned sequences using the BIC criterion [56]. Maximum likelihood (ML) analyses were performed using IQ-TREE under the TVM + F + R5 model [57]. The best-scoring ML tree was generated using 2,000 ultrafast bootstrap replicates. Bayesian inference (BI) phylogenies were inferred using MrBayes v3.2.6 under the GTR + F + G model [58]. Two independent Markov chain Monte Carlo (MCMC) runs were performed with 2,000,000 generations, sampling every 100 generations. An initial 25% of the sampled trees were discarded as burn-in. Posterior probability (PP) values were computed based on the remaining trees.

## Results

### Comparison of morphological and TLC traits

**Fig. 1:**
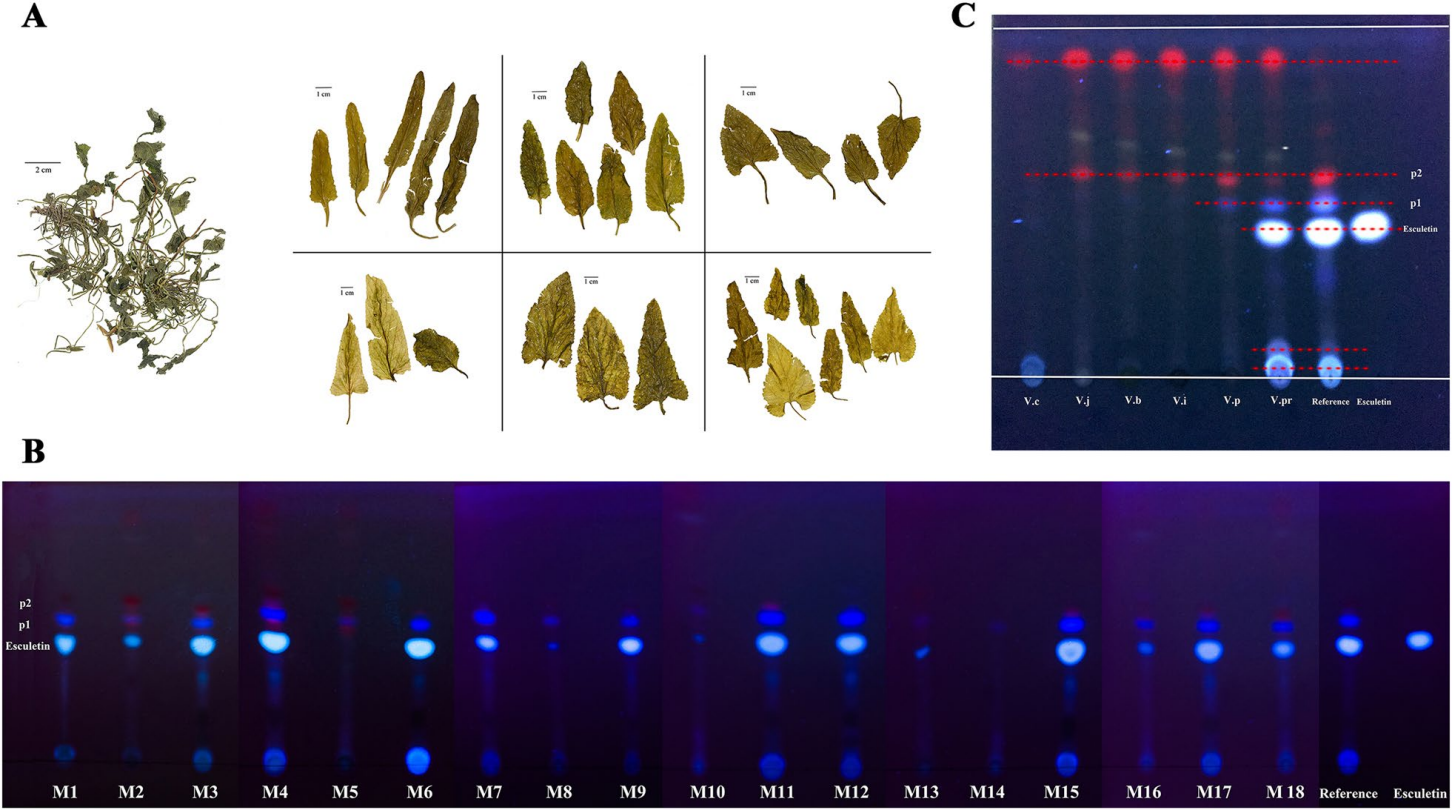
Comparison of morphological and TLC traits. **A** Leaves of the commercial dried samples were expanded in the water to show the morphological traits. Each cell indicates one typical blade shape. **B** TLC traits of 18 batches of Violae Herba with the standard drug and esculetin as references. M1-M18 indicate the 18 batches of samples from the TCM markets. **C** TLC traits of the six *Viola* species with the standard drug and esculetin as references. V.c *V. collina*, V.j *V. japonica*, V.b *V. betonicifolia*, V.i *V. inconspicua*, V.p *V. philippica*, V.pr *V. prionantha*, p1 and p2 are two specific identification spots

Inspection of the dry samples expanded in water showed that the blade shape of commercial VH was variable (Fig. 1A) and not within the variation range of *V. philippica*. Differences in the vegetative period resulted in misidentification of the origin of VH.

To understand the actual plant origin of VH, eighteen samples from TCM markets were inspected and analyzed by TLC (Fig. 1B). Using esculetin as the reference substance together with the standard drug reference [30], we observed that the chromatograms were discrepant. There were clearly three main types of spots. Samples M1, M2, M3, M4, M6, M7, M8, M9, M10, M11, M12, M13, M15, M16, M17, and M18 had almost the same spots as the reference. Samples M2, M8, M10, M13, and M16 contained traces of esculetin but had some of the same spots specific to the reference drug. However, samples M5 and M14 contained almost no esculetin and spots specific to the other two sample types.

**Fig. 2:**
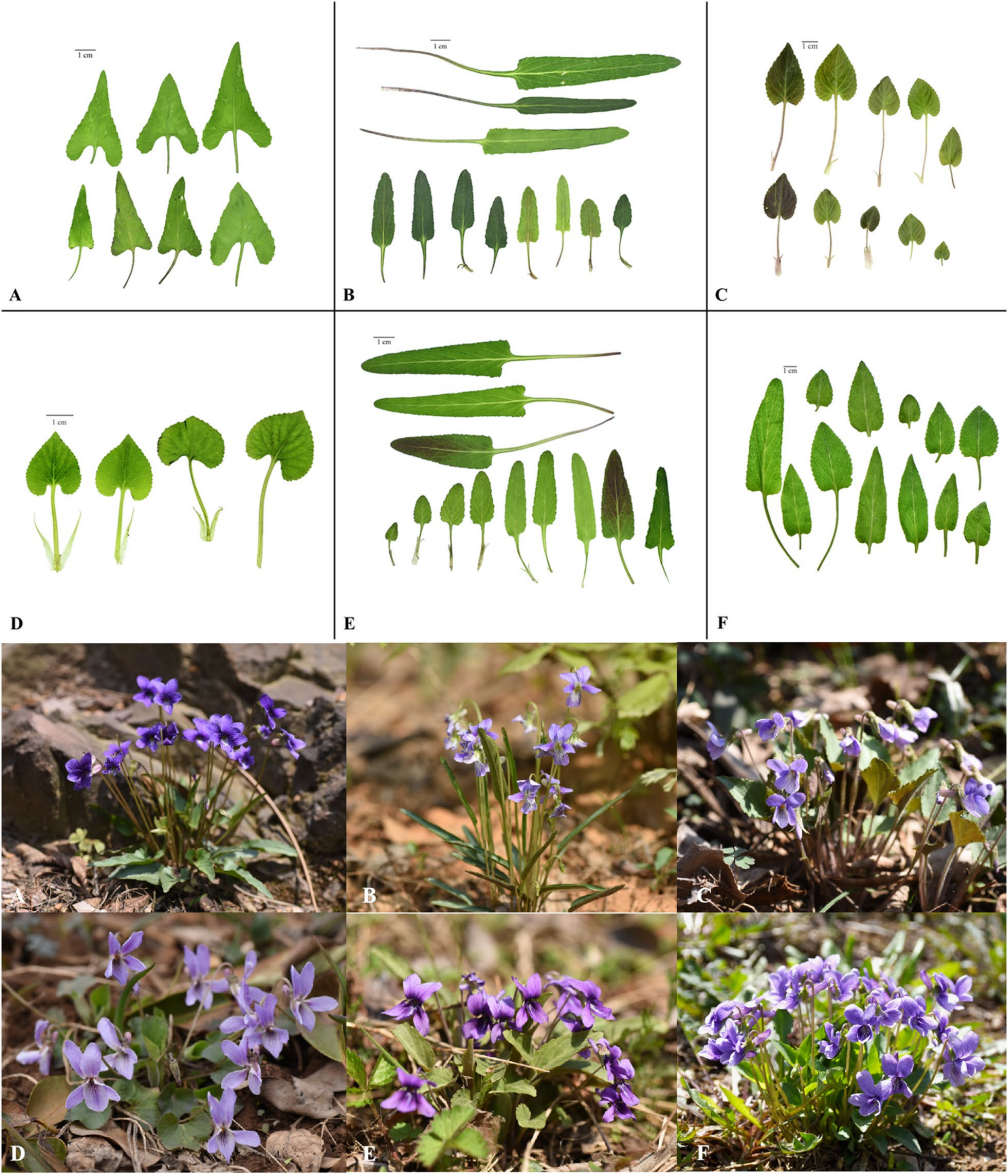
Fresh leaves variation and original plants of the six *Viola* species. **A** *V. inconspicua*; **B** *V. betonicifolia*; **C** *V. japonica*; **D** *V. collina*; **E** *V. philippica*; **F** *V. prionantha*

Commercial VH in TCM markets is sourced mainly by wild collection rather than cultivation. Thus, it has been difficult for herbalists to collect the correct species regulated by the ChP (i.e., *V. philippica*). Based on text- and field-based research, five widespread medicinal plants of the genus *Viola* from southern to northeastern China were collected (Additional file 4: Figures S1, Fig. 2); these plants have long been used to adulterate commercial VH. These species are considered to be the most common homonyms of the Chinese name “Zi Hua Di Ding”. The morphological features were compared with commercial VH for tentative plant origin identification (Fig. 2, Additional file 4: Figures S2, Additional file 1: Table S1).

TLC of these six species was performed (Additional file 2: Table S2). When comparing the TLC results (Fig. 1B, C), we observed that *V. prionantha* (*V.pr*) had the same specific spot as the standard reference. The esculetin spot was not identified in samples other than the *V. prionantha* sample. Thus, most commercial VH samples mainly consist of *V. prionantha* rather than *V. philippica*. These results basically conform to the tentative identification inferences (Additional file 1: Table S1).

### Comparison of characteristic constituents and molecular authentication

Information on the samples used in this section is listed in Additional file 2: Table S2. A total of 42 samples of the six species were collected in different parts of China for the comparison of characteristic constituents. HPLC chromatograms of each species were determined, and characteristic spectra were generated for comparative analysis (Additional file 4: Figure S3). HPLC-Triple-TOF–MS/MS was performed to identify the main constituents.

**Fig. 3:**
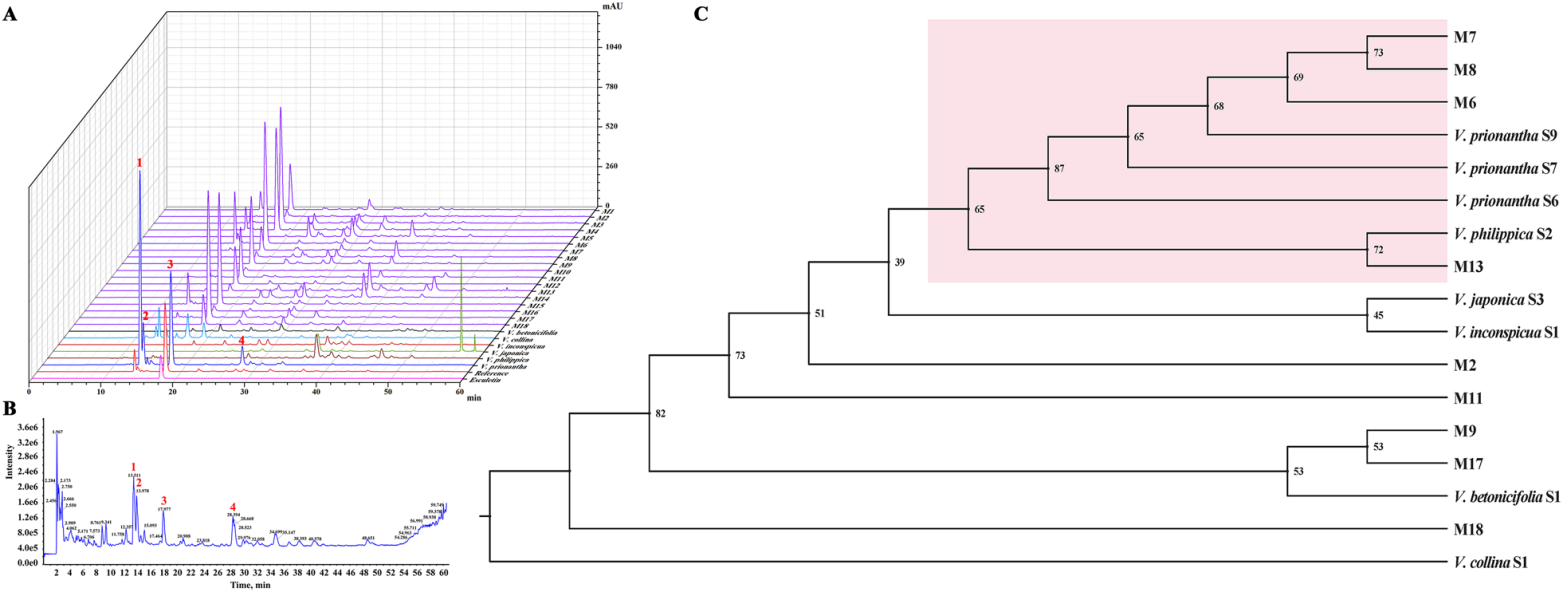
Comparative studies of the index components and universal DNA barcoding authentication. **A** HPLC chromatograms of commercial VH, wild-collected samples, and reference drug. **B** Total ion current chromatogram of *V. prionantha* indicating the 4 main index components in positive ion mode. **C** NJ tree constructed by a two-locus barcode *rbcLa* and *trnH-psbA*

Four main constituents were identified in *V. prionantha*: cichoriin, esculin, esculetin, and prionanthoside. We specified these characteristic constituents as the index components for VH authentication (Additional file 4: Figure S4, Additional file 5: Table S4). Traces of esculin and esculetin were identified in the legal origin *V. philippica* but could hardly be detected in the HPLC chromatogram. These four coumarins and their glycosides were not detected in the other species or had extremely low contents. Most commercial VH and standard reference drugs presented chromatograms almost identical to those of *V. prionantha* (Fig. 3A, B).

The DNA barcoding method was also used to test whether the universal barcodes could be applied to discriminate commercial VH and closely related *Viola* medicinal plants. However, successful PCR amplification of the decoction pieces from TCM markets is relatively difficult due to DNA degradation during postharvest processing [59]. Nine out of the 18 commercial samples produced PCR products for both the *rbcLa* and *psbA-trnH* segments (Additional file 1: Table S1). Together with 8 fresh plant materials, a NJ tree was constructed using two-locus concatenated barcodes (Fig. 3C). Three samples could be clustered with *V. prionantha*, and the majority of the samples identified as *V. prionantha* by morphological and chemical methods could not be distinguished. Although *V. prionantha* could be demonstrated as the mainstream certified origin of VH, it is still difficult to find suitable DNA barcodes for accurate and rapid identification.

In the following sections, we focused on the cp genomes of these six species to gain a deeper understanding of their similarities and differences at the genetic level and to develop more efficient barcodes to authenticate VH and its many impostors.

### General features of chloroplast genomes

**Fig. 4:**
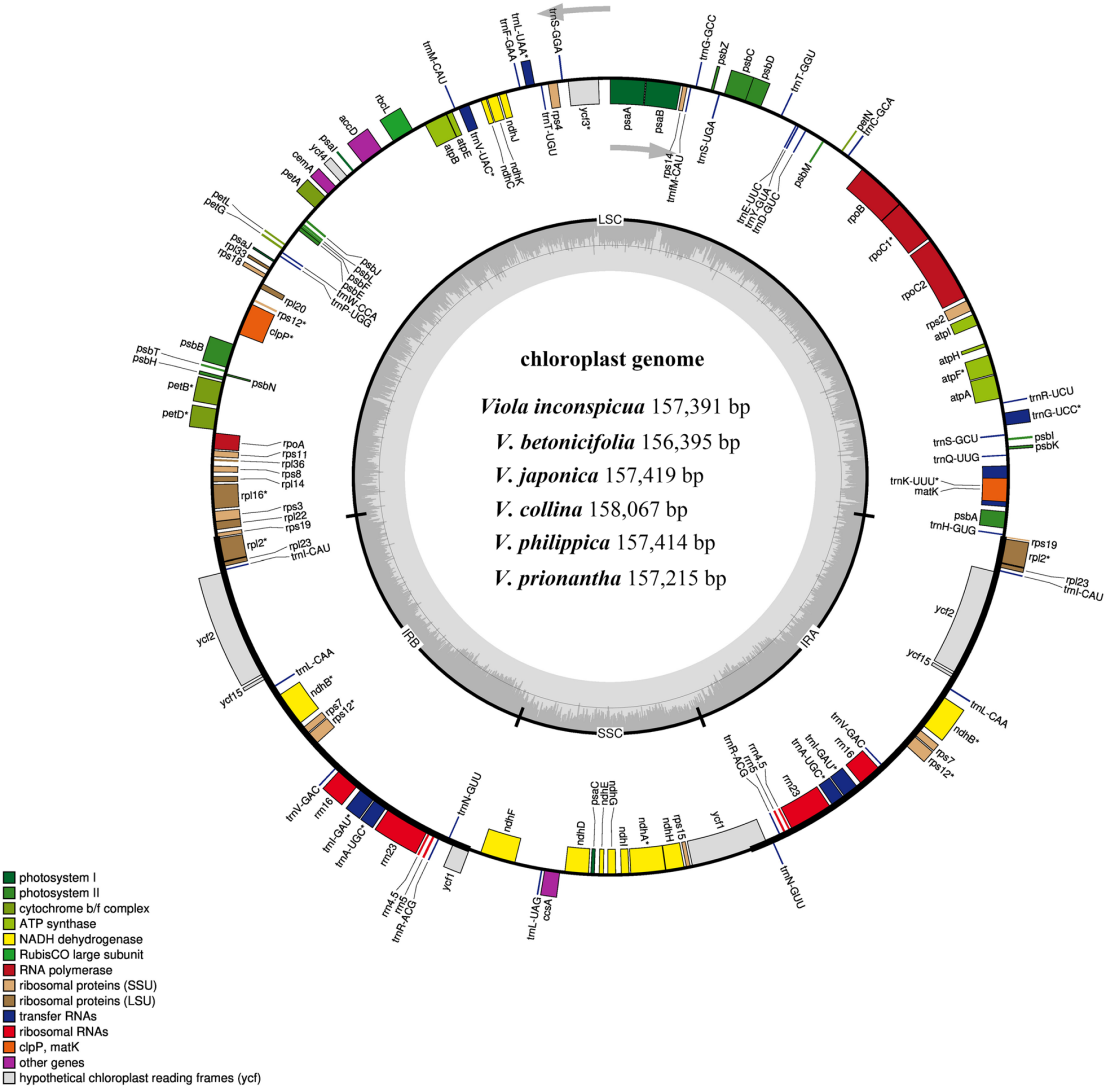
Chloroplast genome map of the six *Viola* species. Only one map was drawn here, because the number of functional genes and linear arrangement are almost the same. Genes drawn inside the circle are transcribed clockwise, genes outside are transcribed counterclockwise. Genes possess different functions are color coded in the legend. The area in darker gray and lighter gray in the inner circle indicates GC content and AT content, respectively

A total of 33,220,638–53,658,183 clean reads were obtained after whole-genome sequencing. The complete chloroplast genome was assembled using mapped reads, with a mapping ratio of 3.94–10.11% (Additional file 3: Table S3). *Viola* plastomes exhibited typical tetrad and circular structures with good synteny (Fig. 4). The genome lengths varied between 156,395 and 158,067, including large and small single-copy regions (LSC: 85,692–86,509 bp, SSC: 16,338–17,330 bp) and a pair of inverted repeat regions (IRa, IRb: 27,105–27,141 bp). The overall GC content of the cp genomes was between 36.24% and 36.35%. The GC contents of the LSC and SSC regions were 33.81–33.87% and 29.76–30.16%, respectively, whereas the IR regions possessed a higher GC content of 42.12–42.16% (Table 1). Four ribosomal RNA (rRNA) genes, which were 55.38% GC (Additional file 6: Table S5), were located in the IR region; this may be one of the factors that made IR regions more conserved than the LSC and SSC regions [60]. The cp genome sequence can be segmented into five zones according to function (Additional file 6: Table S5). The tRNA and rRNA genes of the coding region possessed the highest GC content (> 52%); in contrast, the intergenic spacer of the noncoding region had the lowest GC content (< 30%).

**Table 1:** The basic cp genome features of six *Viola* species

Characteristics	*V. inconspicua*	*V. betonicifolia*	*V. japonica*	*V. collina*	*V. philippica*	*V. prionantha*
Total size (bp)	157,391	156,395	157,419	158,067	157,414	157,215
Overall GC content (%)	36.25	36.35	36.25	36.25	36.24	36.25
LSC						
Length (bp)	85,828	85,779	85,908	86,509	85,907	85,692
GC content	33.83	33.87	33.82	33.82	33.81	33.83
Length (%)	54.53	54.85	54.57	54.73	54.57	54.51
IR						
Length (bp)	27,161	27,139	27,116	27,114	27,118	27,105
GC content (%)	42.12	42.14	42.14	42.16	42.14	42.15
Length (%)	17.26	17.35	17.23	17.15	17.23	17.24
SSC						
Length (bp)	17,241	16,338	17,279	17,330	17,271	17,313
GC content (%)	29.79	30.16	29.83	29.91	29.76	29.76
Length (%)	10.95	10.45	10.98	10.96	10.97	11.01
Genes						
Total genes	129	129	129	129	129	129
Duplicate genes	18	18	18	18	18	18
Protein coding genes	77	77	77	77	77	77
rRNA genes	4	4	4	4	4	4
tRNA genes	30	30	30	30	30	30

*GC* guanine and cytosine, *LSC* large single-copy region, *SSC* small single-copy region, *IRs* inverted repeats region

A total of 129 genes were annotated from these cp genomes, including 84 protein-coding genes, 8 rRNA genes, 37 tRNA genes, and two pseudogenes (Ψ*ycf1* and Ψ*rps19*). Eighteen of them were duplicated in the IR regions, including seven protein-coding genes (*rps7*, *rps12*, *rpl2*, *rpl23*, *ndhB*, *ycf2*, and *ycf15*), seven tRNA genes (*trnA*-*UGC*, *trnI*-*CAU*, *trnI*-*GAU*, *trnL*-*CAA*, *trnN*-*GUU*, *trnR*-*ACG*, and *trnV*-*GAC*), and four rRNA genes (*rrn16*, *rrn23*, *rrn4.5*, and *rrn5*) (Table 2, Additional file 7: Table S6). The protein-coding gene *rps12* is a trans-spliced gene with its 5’ terminus located in the LSC region and its 3’ terminus having a copy located in each of the IR regions in two independent transcription units. Except for the genes located in the IR boundary, four pairs of overlapping genes were identified. The *matK* gene was included in the *trnK* intron; *psbD* and *psbC* had a 53-nt overlap; *atpE* and *atpB* and *rps3* and *rpl22* overlapped by 4 nt and 16 nt, respectively. However, a complete loss of the *infA* and *rps16* genes in the LSC and the *rpl32* gene in the SSC was revealed, which coincided with most other species in Malpighiales [61, 62].

**Table 2:** Gene contents in the cp genomes of six *Viola* species

Group of genes	Name of genes	Amount
Ribosomal RNAs	rrn16(× 2), rrn23(× 2), rrn4.5(× 2), rrn5(× 2)	8
Transfer RNAs	trnA-UGC*(× 2), trnC-GCA, trnD-GUC, trnE-UUC, trnF-GAA, trnG-GCC, trnG-UCC*, trnH-GUG, trnI-CAU(× 2), trnI-GAU*(× 2), trnK-UUU*, trnL-CAA(× 2), trnL-UAA*,trnL-UAG, trnfM-CAU, trnM-CAU, trnN-GUU(× 2), *trnP-UGG, trnQ-UUG, trnR-ACG*(× 2), *trnR-UCU, trnS-GCU, trnS-GGA, trnS-UGA, trnT-GGU, trnT-UGU, trnV-GAC*(× 2), trnV-UAC*, trnW-CCA, trnY-GUA	37
Small ribosomal subunit (SSU)	*rps2, rps3, rps4, rps7*(× 2), rps8, rps11, rps12**(× 2), *rps14, rps15, rps18, rps19*	13
Large ribosomal subunit (LSU)	rpl2*(× 2), rpl14, rpl16*, rpl20, rpl22, rpl23(× 2)*, rpl33, rpl36*	10
DNA-dependent RNA polymerase	rpoA, rpoB, rpoC1*, rpoC2	4
Subunits of ATP synthase	atpA, atpB,atpE, atpF*, atpH, atpI	6
Subunits of NADH dehydrogenase	ndhA*, ndhB*(× 2), *ndhC, ndhD, ndhE*, *ndhF, ndhG, ndhH, ndhI, ndhJ, ndhK*	12
Cytochrome b/f complex	petA, petB*, petD*, petG, petL, petN	6
Subunits of photosystem I	*psaA, psaB, psaC, psaI, psaJ*	5
Assembly/Stability of photosystem I	ycf3**, ycf4	2
Subunits of photosystem II	*psbA, psbB, psbC, psbD, psbE, psbF, psbH, psbI, psbJ, psbK, psbL, psbM, psbN, psbT, psbZ*	15
Large subunit of Rubisco	*rbcL*	1
Acetyl-CoA carboxylase	*accD*	1
c-type cytochrome synthesis gene	*ccsA*	1
Envelop membrane protein	*cemA*	1
ATP-dependent protease	clpP**	1
Maturase	*matK*	1
Conserved open reading frames	*ycf1, ycf2*(× 2)*, ycf15*(× 2)	5
Pseudogenes	^*Ψ*^*ycf1, *^*Ψ*^*rps19*	
Total		129

One and two asterisks (*) indicate one- and two-intron containing genes. Genes with two copies indicate by the (× 2) symbol. (Ψ) symbol indicates the pseudogene. The *rps12* gene is a trans-spliced gene

Introns tend to accumulate more mutations than exons and play an important role in the regulation of gene expression [63]. Seventeen genes harbored introns. Fifteen genes contained one intron, and two (*ycf3* and *clpP*) contained two introns (Additional file 8: Table S7).

RNA editing occurs widely throughout plant lifecycles and participates in plastid transcriptional regulation [64]. RNA editing in chloroplasts regulates gene expression and produces different proteins, thus enriching the genetic information [65]. In this study, 34 protein-coding genes in the six *Viola* cp genomes were analyzed for potential RNA editing sites. A total of 57 RNA editing sites were identified, mainly in the *ndh* and *rpo* gene complexes (Additional file 4: Figure S5). Among them, 17 were edited at the first position of the corresponding codon, and 40 were edited at the second position. Transitions in the third codon position were not observed; moreover, the base conversion type was C to U (Additional file 9: Table S8). The substitution of serine and proline residues by leucine occurred most frequently; hence, the primary structure of the protein became more hydrophobic. In general, these results are similar to those for other land plants [66].

### Codon usage bias

Codon usage bias can be used to investigate evolutionary processes based on genomes at the molecular level [67]. The GC content of the third codon position (GC3) reflects directional mutation pressure and is closely related to codon bias [68]. Codon usage patterns of CDS regions were calculated based on their relative synonymous codon usage (RSCU) values. In this study, the numbers of codons in the CDS region were approximately 26,297–26,323, with 84 coding sequences for each of the six species. The GC3 contents (29.25–29.47%) were lower than those at the first (44.92–44.97%) and second (37.40–37.48%) positions (Additional file 10: Table S9). AT preference in the third position in the cp codons also appears in other angiosperms [69].

**Fig. 5:**
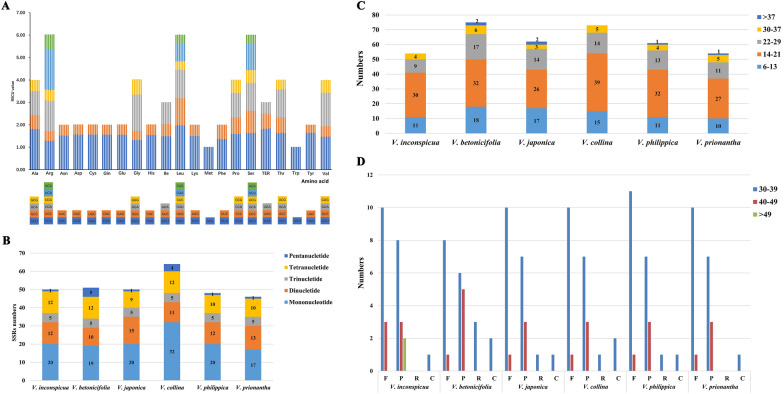
Comparative analyses of codon usage and repeat sequences. **A** Codon contents for the CDS sequences in the six cp genomes. The ordinate shows the RSCU values, and the abscissa represents 20 amino acids and termination codons. The histogram represents codon usage for each amino acid of the six species (From left to right: *V. inconspicua*, *V. betonicifolia*, *V. japonica*, *V. collina*, *V. philippica*, *V. prionantha*). **B** Types and numbers of SSRs **C** Numbers of tandem repeats. **D** Types and numbers of dispersed repeats. F, P, R, and C indicate the repeat types F(forward), P(palindrome), R(reverse), and C(complement)

Among the coding sequences of the six species, 2794–2808 codons encoded leucine and 300–305 encoded cysteine, which are the most and least abundant amino acids, respectively (Additional file 4: Figure S6). The RSCU analysis of the cp genomes of the six *Viola* species is shown in Additional file 11: Table S10 and Fig. 5A. The RSCU values only differed slightly from each other, with total values of 1–6%. The effective number of codons (ENC) was approximately 49.4 in all six species, indicating a slight bias in codon usage.

### SSR and long repeat analyses

SSRs, also known as DNA microsatellites, are short (1–6 bp), tandemly repeated DNA sequences distributed throughout the cp genome [70]. Other than those of SSRs, the lengths of the tandem repeat motifs were > 6 bp; repeats dispersed in genomes with lengths ≥ 30 bp are called long repeat sequences. Long tandem repeats and dispersed repeats, including forward repeats (F), palindromic repeats (P), reverse repeats (R), and complement repeats (C), play an important role in the rearrangement of the cp genome and increase the genetic diversity of a population [71].

In total, between 46 (*V. prionantha*) and 64 (*V. collina*) SSRs were identified in the six *Viola* species. Among the SSRs, mononucleotide repeats occurred most frequently, followed by dinucleotide and tetranucleotide repeats (Fig. 5B). More than 90% of SSRs contained short poly(A) and poly(T) repeats, which resulted in a bias toward A/T usage in the genome. In addition, these SSRs were mainly distributed in the intergenic spacer (IGS) and LSC regions, which contained 66.77% and 69.47% of the SSRs, respectively (Additional file 12: Tables S11–S13).

Long motifs of tandem and dispersed repeats were also identified in these six *Viola* species (Fig. 5C, D). In *V. inconspicua* and *V. prionantha*, 54 tandem repeats were detected, and in *V. betonicifolia*, 75 tandem repeats were detected. The lengths of the tandem motifs were generally less than 29 bp (91.28%) and mostly within 14–21 bp (49.34%). Four types of dispersed repeats were also detected, and their total numbers were slightly different. However, no reverse repeat was found in *V. inconspicua* and *V. prionantha*, whereas three were found in *V. betonicifolia* and only one was found in the other three species. In addition to the 1–2 complement repeats, forward and palindromic repeats constituted the majority of the dispersed repeats (90.37%). Most ranged in size from 30 to 39 bp.

The number of long repeat types was similar among the six cp genomes, and their distribution in the plastome was highly conserved, which is summarized in Additional file 13: Table S14 and Additional file 14: Table S15. Long tandem repeats were mainly distributed in the IGS regions of LSCs (75.04%). Dispersed repeats were discretely distributed in the LSC and IR regions, and only the dispersed repeats in the *ndhA* intron were located in the SSC. The mining of cp SSRs and long repeat markers with high substitution rates can be applied to further evolutionary, genetic diversity, and species identification studies of *Viola*.

### Sequence divergence and hotspots

**Fig. 6:**
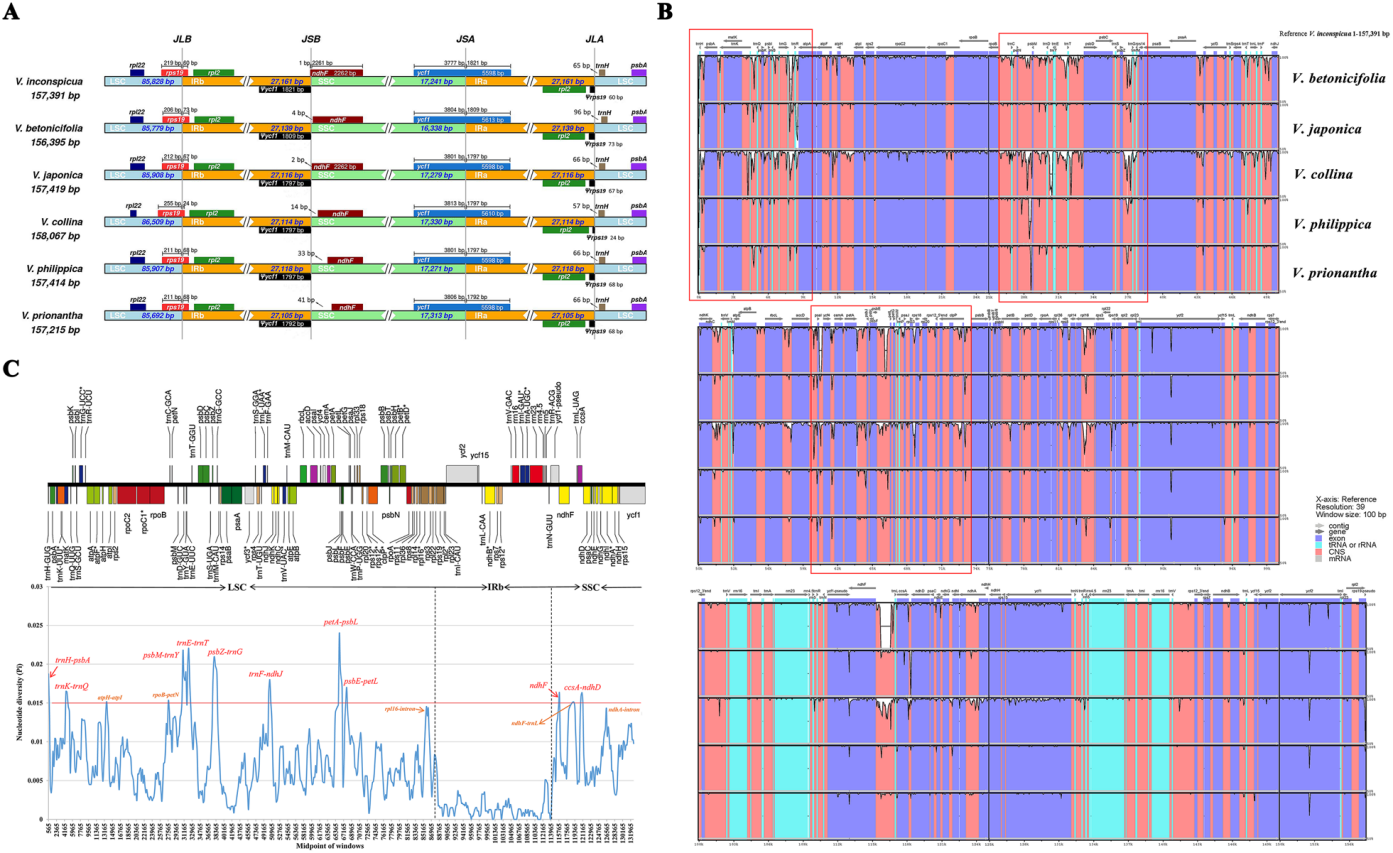
Analyses of sequences divergence. **A** Comparison of the LSC, IR, and SSC junctions. Colored boxes for genes represent the gene position. JLB denotes the LSC/IRb junction, JSB denotes the SSC/IRb junction, JSA denotes the SSC/IRa junction, and JLA denotes the LSC/IRa junction. **B** Global alignment of six *Viola* cp genomes using the annotation of *V. inconspicua* as a reference. Dark blue blocks indicate exons, and red blocks indicate conserved non-coding sequences (CNS). tRNA and rRNA genes are denoted by cyan blocks. White peaks represent regions with sequence variation among cp genomes. Arrows are drawn above the graph pointing the direction of genes. 70% cut-off was used for the plot, and the vertical axis indicates sequence identity between 50 and 100%. The red rectangle frames indicate the divergent hotspot regions. **C** Sliding window analysis of the complete cp genomes of six *Viola* species (window length: 800 bp, step size: 200 bp). X-axis indicates the midpoint of windows. Y-axis indicates nucleotide diversity (Pi) within each window. And the red line indicates the threshold for potential genetic markers (Pi threshold = 0.015). Regions with Pi values meet or exceed 0.015 were marked out on the graph. The multicolored legend above shows the gene order

The contraction and expansion of IR regions result in length variations in the cp genomes in angiosperms [72]. Hence, the IR/SC boundary regions of the six *Viola* cp genomes were compared. The beginning of each genome was aligned and standardized to be the first base pair immediately after the IRa region. We found that all six *Viola* cp genomes had highly conserved IR borders (Fig. 6A). The SSC/IRa boundary of all species was located in the *ycf1* gene, resulting in the duplication of the *ycf1* pseudogene in the IRb region, and the LSC/IRb junction expanded into the *rps19* gene.

To further characterize divergence at the genomic level, global sequence alignments were used to investigate polymorphic regions in *Viola* cp genomes. As indicated in Fig. 6B, the LSC and SSC regions were more divergent, and the IR regions were the most conserved. The majority of the variable sites were found in intergenic spacers; thus, noncoding regions, including some introns (e.g., *rpl16* and *ndhA*), were much more divergent than coding regions. According to the mVISTA program output, three divergent hotspot regions were found in the IGS regions between flanking genes *trnH*-*atpA*, *rpoB*-*rps14*, and *accD*-*psbB* (Fig. 6B). These results indicate that these regions evolve rapidly in the genus *Viola*, as well as in other related Malpighiales species [73].

Nucleotide diversity (Pi) was used to identify hypervariable regions. Specific segments in hotspot regions could be further utilized as potential genetic markers when combined with sliding-window analysis of nucleotide diversity and sequence polymorphisms for the six *Viola* cp genomes.

The average Pi value for the six *Viola* cp genomes was 0.00649, and the IR regions showed lower nucleotide diversity than the single-copy regions. The Pi value for the noncoding regions was much higher than that for the CDS regions (Additional file 15: Table S16). Additionally, 15 noncoding segments from the LSC/SSC regions showed a relatively higher Pi than other regions and were therefore considered hypervariable regions (Fig. 6C). These hypervariable regions included 12 IGS regions (*trnH-psbA*, *trnK-trnQ*, *psbM-trnY*, *trnE-trnT*, *psbZ-trnG*, *trnF-ndhJ*, *petA-psbL*, *psbE-petL*, *ccsA-ndhD*, *atpH-atpI*, *rpoB-petN*, and *ndhF-trnL*), a protein-coding region (*ndhF*), and two introns (*rpl16* and *ndhA*), which is in line with the mVISTA analysis.

As shown in Additional file 15: Table S16, the *trnH*-*psbA* region showed the highest diversity and served as one of the traditional DNA markers [13]. The next most variable regions were *petA*-*psbJ*-*psbL*, *ccsA*-*ndhD*, and *psbZ*-*trnG*, while the diversity of *ndhF* was the lowest. Four of these screened regions have been reported to be potential hypervariable markers in *Viola: trnH*-*psbA*, *psbZ*-*trnG*, *petA*-*psbJ*, and *ndhF*-*trnL* [29]. Several universal cp DNA markers have been used in phylogenetic studies of *Viola* or Violaceae, such as *trnL*-*trnF*, *trnH*-*psbA*, *rpl16*-intron [74], *rbcL* [75], *matK*, *atpB*-*rbcL*, *atpF*-*atpH*, *psbK*-*psbI*, and the second exon of *rpoC1* [16]. However, the nucleotide diversity of these conventional markers was relatively lower than that of the hypervariable regions, except for *psbK*-*psbI*, *atpF*-*atpH*, and *trnH*-*psbA*. As a consequence, the use of markers with low Pi values in previous studies led to low phylogenetic resolution and discrimination ability [76]. The highly variable regions found here are more conducive for species authentication and phylogenetic studies.

### Phylogenetic relationships

**Fig. 7:**
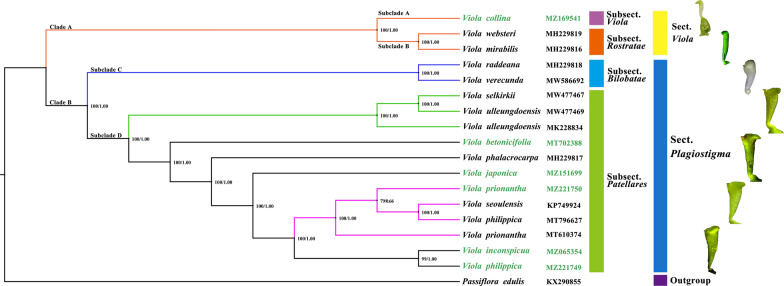
Phylogenetic tree based on the cp genomes of 17 *Viola* species using maximum likelihood (ML) and Bayesian inferences (BI) methods. *Passiflora edulis* was used as outgroup. Numbers besides the nodes represent ML bootstrap support (BS) values and BI posterior probabilities (PP) values. The legend on the right shows the Section-Subsection taxonomic synopsis and the corresponding evolution of stigma

In this study, the cp genomes of six newly sequenced species, along with the 11 complete cp genome sequences of *Viola* downloaded from GenBank (*Passiflora edulis* as outgroup), were used to construct the *Viola* infrageneric phylogeny (Additional file 16: Table S17). The trees inferred from the ML and BI methods revealed the same topology (Fig. 7). All these species are classified into Subgenus *Viola* in the Flora of China (FOC). However, the phylogenetic tree indicated that the Subgenus-Section taxonomic synopsis was not valid at the infrageneric level. Clades A and B were the two main parallel clades, representing two evolutionary directions (Fig. 7). The direct use of the Section-Subsection synopsis can provide a more explicit phylogenetic framework and can reflect the evolutionary trend in the morphology of the stigma.

### Species authentication and development of plant superbarcodes

The low Pi values and lack of informative sites demonstrated the limitation of current universal DNA barcodes (*matK*, *trnL*-*trnF*, *rbcL*, *atpB*-*rbcL*, and *rpoC1* exon 2) and their combinations in distinguishing *Viola* species at the sectional and species levels [16]. Hypervariable regions identified by the comparison of complete cp genomes offer adequate resources to develop specific new cp DNA barcodes to discriminate between *Viola* species. Among the hotspot regions and universal barcodes, seven intergenic regions with Pi values greater than 0.02 were selected as potential cp DNA barcodes, along with two conventional markers, *psbK*-*psbI* and *atpF*-*atpH*, which were relatively more variable than other universal barcodes (Table 3).

**Table 3:** The variability and discrimination rates of cp genomes, specific and universal barcodes

No	Markers	Aligned length	Polymorphic sites		Parsimony informative sites (PICs)		Mean K2P distance	Discrimination rates by tree-based method
			Numbers	%	Numbers	%		
	Complete cp genomes	136,352	4664	3.42%	2175	1.60%	0.00824	100%
	Specific barcodes							
1	*trnH(GUG)-psbA*	506	65	12.85%	30	5.93%	0.03880	100%
2	*trnE(UUC)-trnT(GGU)*	1155	118	10.22%	58	5.02%	0.02759	35.29%
3	*psbZ-trnG(GCC)*	1189	138	11.61%	55	4.63%	0.03028	64.71%
4	*trnF(GAA)-ndhJ*	977	157	16.07%	108	11.05%	0.04316	70.59%
*5*	*petA-psbL*	1580	129	8.16%	69	4.37%	0.03071	64.71%
6	*ccsA-ndhD*	300	46	15.33%	24	8.00%	0.04475	41.18%
7	*ndhF-trnL(UAG)*	1923	222	11.54%	93	4.84%	0.03428	47.06%
	Combination (1–2-3)	2850	321	11.26%	143	5.02%	0.03034	100%
	Universal barcodes							
1	*psbK-psbI*	424	25	5.90%	17	4.01%	0.02121	41.18%
2	*atpF-atpH*	625	46	7.36%	15	2.40%	0.01793	47.06%
	Combination (1–2)	1049	71	6.77%	32	3.05%	0.01929	47.06%

Discrimination rates (%) = [the number of species with bootstrap values more than 50% / the total number of species] × 100%

It is imperative to compare rapidly evolving markers with conventional markers for feasibility in *Viola* species identification. Hence, these potential barcodes and the whole cp genomes were tested for their discrimination ability within 11 more closely related *Viola* species (Additional file 16: Table S17). The discrimination rates of the specific and universal barcodes were compared using NJ tree-based methods (Additional file 17: Figures S7). If the bootstrap value of the node was less than 50, suggesting low branch support, then species on that branch were not counted [77].

**Fig. 8:**
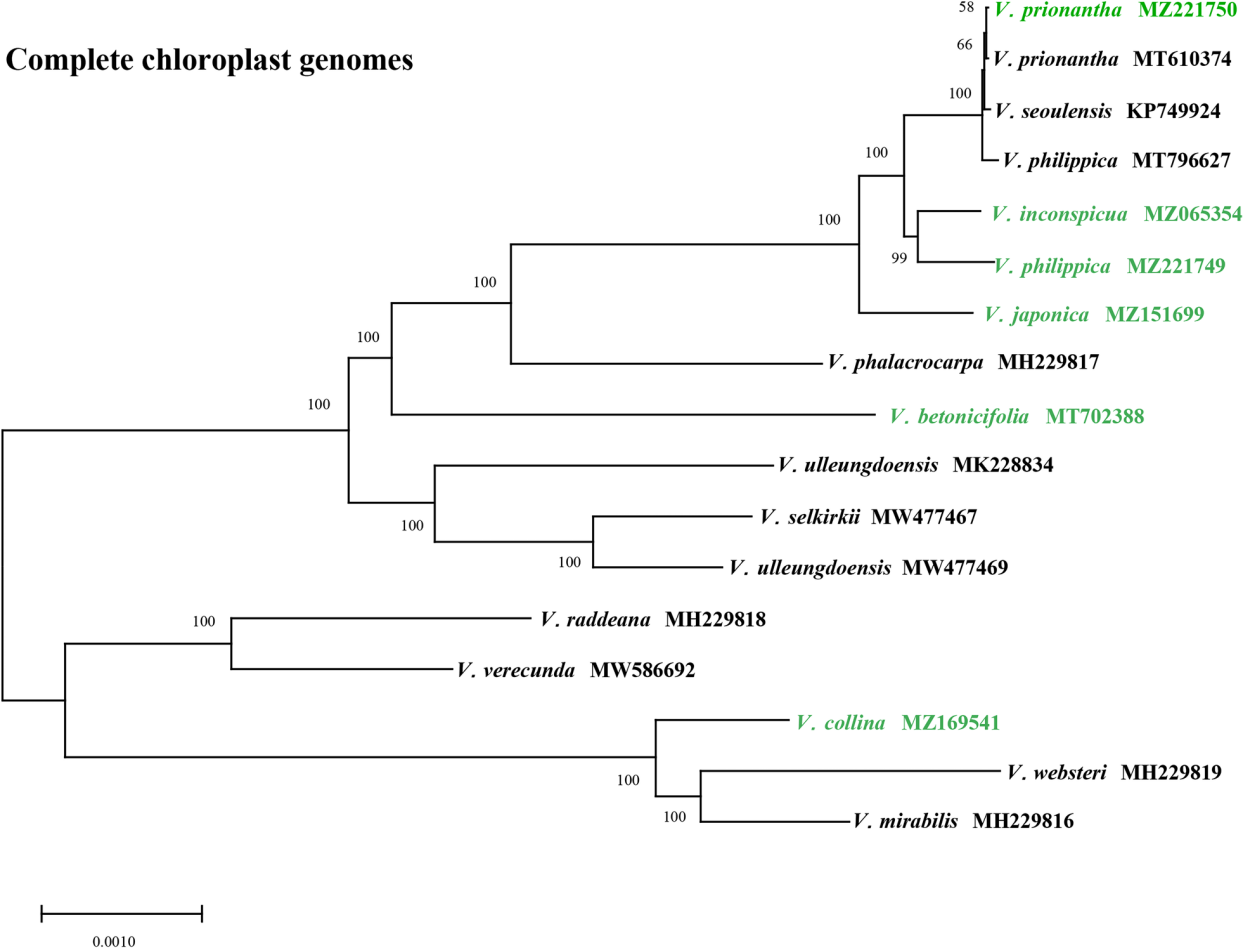
Neighbor-joining tree for 17 *Viola* species using complete cp genomes as super-barcodes

Four out of the seven selected specific barcodes showed high discrimination rates of more than 50%. The two universal barcodes and their combination had low species identification ability (Additional file 18: Figures S8). Moreover, the NJ trees constructed by these barcodes should have a similar topology as the phylogenetic tree inferred by the complete cp genome (Fig. 7). Based on the tree topology and identification rates, three intergenic regions, *trnH-psbA*, *trnE-trnT*, and *psbZ-trnG,* were concatenated as a combined barcode. The complete cp genome as a “superbarcode” and the three-locus concatenated barcode have a potential discrimination power of up to 100% (Fig. 8, Additional file 18: Figures S8), but the latter had relatively low branch support.

Compared with the single- or multi-locus cp DNA barcode, the complete cp genome as a superbarcode was an efficient and reliable tool to authenticate the original species of VH and congeneric species. As shown in Fig. 8, *V. collina* had the longest genetic distance from the other five species, and the two *V. prionantha* materials were clustered together with *V. seoulensis* [78]. The reported *V. philippica* was not clustered with the newly sequenced material but rather clustered with *V. prionantha* [79]. In addition, this previously reported *V. philippica* sample was collected in Qinghai Province, where no distribution of this species was recorded. Thus, this species may be misidentified as *V. prionantha*. The name *V. seoulensis* has not been established as an accepted name and was cited as a synonym of *V. phalacrocarpa*, which was demonstrated in a previous study [29]. However, comparative studies of cp genomes have revealed that this species is closely related to *V. prionantha*.

## Discussion

Violae Herba was found to be a multisource crude drug in the current TCM markets. The certified source of VH is undefined, and multiple origins make it difficult to guarantee its quality and medication safety. Comparative studies suggested that *V. prionantha* is the main plant source of VH. The legal origin *V. philippica* contains very little esculetin and thus could not meet the ChP standards (Fig. 3A). Moreover, even the National Drug Reference Standard of Violae Herba (No. 121429) from the NIFDC is made from *V. prionantha*, as inferred from the TLC and chemical constituent results (Fig. 1C and 3A). Reference standards are the essential tool and material basis for drug quality control [80]. An incorrect standard may create increased confusion regarding VH.

Homonyms and synonyms are major problems that lead to the confusion regarding VH [81]. On the one hand, the name recorded in the ChP is *V. yedoensis*. However, it is considered synonymous with *V. philippica* in plant taxonomy monographs. On the other hand, the Chinese name for *V. philippica* is “Zi Hua Di Ding”, which exactly matches the Chinese name for VH. Therefore, initially, we may take it for granted that they refer to the same plant. Furthermore, homonyms of “Zi Hua Di Ding” among populations make it more complicated to clarify the certified origin of VH.

Discrepancies in the chemical constituents and pharmacological effects between *V. philippica* and its adulterants have been reported in previous studies [6–8]. These studies indicated that coumarin and its derivatives are the main active ingredients of VH due to their anticoagulant, antibacterial, and antioxidant activities [82–84]. Four coumarins were identified according to the previous studies of our research group [6, 85] and were specified as the index components for VH authentication. However, outside of *V. prionantha*, the main index components could not be detected or had low contents in *Viola philippica* and other adulterants, as reported in this study. VH is a TCM for clearing heat and detoxification. As a consequence, the source of previous studies on VH is actually *V. prionantha*. The commercial VH and NIFDC reference drugs mostly consist of *V. prionantha* in the current TCM markets. We suggest that the origin of VH in the ChP should be revised to *V. prionantha* (Chinese name: Zao Kai Jin Cai), and the item name in ChP should remain “Zi Hua Di Ding”.

Though the certified origin of VH can be inferred from comparative studies of TCM market investigations, morphology, and its main chemical constituents, it remains difficult to maintain as a single-source medicine. Conventional methods are not reliable enough to distinguish samples with qualified origins from several adulterants. Given the demand for accurate and rapid authentication of often-confused *Viola* species, DNA barcoding has been proposed as an effective tool. By using one or several standardized DNA regions as universal barcodes, numerous multisource TCMs have been successfully identified [86]. However, the discriminative ability of universal barcodes was low for closely related or recently evolved species [87], owing to the lack of informative sites.

Herbgenomics plays an important role in the modernization of TCM, and molecular identification by DNA barcoding has reached the genomic level [88, 89]. With the rapid development of NGS, cp genome sequences can be obtained efficiently and economically [90]. It is possible to develop new barcodes derived from the cp genomes or use the whole cp genome as a superbarcode to authenticate species-rich genera [25–27]. cp DNA markers were more commonly used in phylogenetic studies of *Viola* rather than species authentication studies [15, 16, 74, 76]. In this study, the concatenated regions *trnH-psbA*, *psbZ-trnG*, and *trnE-trnT* were developed as a cp DNA barcode that has relatively strong species authentication ability. The hypervariable regions *trnH-psbA* and *psbZ-trnG* have been investigated in previous studies [29]. The *trnE-trnT* intergenic region has also been tested as a potential DNA barcode in other taxa [91, 92]. All cp genomes as superbarcodes were also developed to evaluate the feasibility of discriminating closely related species of *Viola*.

The results of our study suggested that the NJ trees constructed by complete cp genomes and three-locus concatenated barcodes showed a high discrimination ability for the *Viola* species (Additional file 18: Figure S8, Fig. 8). The cp genomes used as superbarcodes presented an identical topology as the phylogenetic tree constructed by ML and BI methods and had the highest support values. However, the whole cp genome sequence is difficult to obtain from commercial TCM because of cp DNA degradation. Hence, the specific barcodes and cp genome barcodes are equally important. More work should be focused on the versatility of these barcodes as complementary methods applicable to diverse authentication demands.

Moreover, the pharmacophylogenetic relationships of these plants also need to be further understood. Pharmacophylogeny theory proposes that morphological characteristics, chemical markers, and DNA markers are better combined when making an inference of medicinal plant phylogeny [93]. Medicinal plants with close phylogenetic relationships should have similar chemical components. Integrated approaches in our study have indicated that the origin of VH and its adulterants all belong to the subsection *Patellares* (Fig. 7). *V. prionantha* is not similar to *V. philippica* or other congeneric species based on chemical or genetic results. Our study further clarifies that *Viola prionantha* and *V. philippica* are not alternatives to each other. The only certified origin of VH in the ChP is *Viola prionantha*, while others can only be considered adulterants.

## Conclusions

In this paper, we report comparative analyses of commercial Violae Herba with its plant origin and adulterants. The mainstream plant origin of VH in the current TCM markets is considered to be *V. prionantha* and not *V. philippica*. The complete cp genomes of *V. philippica* and its five adulterants were sequenced and analyzed for the purpose of developing more efficient DNA barcodes for *Viola* species authentication. First, we compared the basic genome features, codon usage bias, repeat sequences, and IR boundaries of these six cp genomes, which showed little difference from each other. Then, when we compared our sequences with more published congeneric species, three specific cp DNA barcodes (*trnH*-*psbA*, *trnE*-*trnT*, and *psbZ*-*trnG*) and their combination were identified as potential DNA barcodes for *Viola*. We also discussed the application of the whole cp genome as a superbarcode to authenticate closely related *Viola* species. Additionally, we propose that the legal origin of Violae Herba in the Chinese Pharmacopoeia should be explicitly certified and revised to *Viola prionantha*.

## Supplementary Information

**Additional file 1: Table S1**. Information of 18 batches of commercial samples and tentative identification.

**Additional file 2: Table S2**. Sample information of *Viola philippica* and its adulterants used for comparative studies.

**Additional file 3: Table S3**. Sample information of six *Viola* species for cp genome sequencing in this study.

**Additional file 4: Figure S1**. The distribution schematic of the six *Viola* species. **Figure S2**. Leaf morphology of the 18 commercial VH for tentative identification. **Figure S3**. The HPLC chromatograms of the wild-collected samples of 6 *Viola* species. **Figure S4**. The total ion current chromatograms of six *Viola* species in negative and positive ion mode. **Figure S5**. The distribution of RNA editing sites. **Figure S6**. Codon numbers for each amino acid of the six *Viola* species.

**Additional file 5: Table S4**. Main chemical components determined by HPLC-Triple-TOF-MS/MS.

**Additional file 6: Table S5**. The basic features of functional regions of cp genomes of six *Viola* species.

**Additional file 7: Table S6**. Genes contained in each part of the cp genomes of six *Viola* species.

**Additional file 8: Table S7**. The lengths of exons and introns in the cp genomes of six *Viola* species.

**Additional file 9: Table S8**. RNA editing sites of the cp genomes of six *Viola* species.

**Additional file 10: Table S9**. The GC content in different positions of codons of six *Viola* species.

**Additional file 11: Table S10**. Codon-anticodon recognition patterns and codon usage of cp genomes of six *Viola* species.

**Additional file 12: Table S11**. Motif types and amounts of SSRs in six *Viola* species. **Table S12.** SSR numbers in different parts of the six *Viola* species. **Table S13**. The distribution of SSRs in the cp genomes of six *Viola* species.

**Additional file 13: Table S14**. Tandem repeat sequences of six *Viola* species.

**Additional file 14: Table S15**. Dispersed repeat sequences of six *Viola* species.

**Additional file 15: Table S16**. Sequence polymorphism of divergent regions and conventional cp DNA markers in *Viola*.

**Additional file 16: Table S17**. The 12 complete chloroplast genome sequences downloaded from GenBank.

**Additional file 17: Figure S7**. NJ trees constructed by 7 variable regions and 2 universal barcodes.

**Additional file 18: Figure S8**. NJ trees constructed by the two-locus universal barcode and three-locus specific barcode.

## Data Availability

The datasets generated during the current study are available in the National Center for Biotechnology Information database (NCBI) under the Bioproject No. PRJNA636230 including all the Biosample and Accession Numbers. The raw data have been deposited in NCBI Sequence Read Archive (SRA, https://www.ncbi.nlm.nih.gov/sra/).

## Abbreviations

VH: Violae Herba
ChP: Pharmacopoeia of the People’s Republic of China (Chinese Pharmacopoeia)
TCM: Traditional Chinese Medicine
cp: Chloroplast
NGS: Next-generation sequencing
TLC: Thin layer chromatography
NIFDC: National Institute for Food and Drug Control (China)
HPLC–DAD: High-performance liquid chromatography coupled with diode array detector
HPLC-Triple-TOF–MS/MS: High performance liquid chromatography triple time-of-flight mass spectrometry
ENC: Effective number of codons
SSRs: Simple sequence repeats
Pi: Nucleotide diversity
PICs: Parsimony informative sites
ML: Maximum likelihood
BI: Bayesian inference
MCMC: Markov chain Monte Carlo
PP: Posterior probability
LSC: Large single copy
SSC: Small single copy
IR: Inverted repeat
RSCU: Relative synonymous codon usage
IGS: Intergenic Spacer
FOC: Flora of China
BS: Bootstrap support
SRA: Sequence Read Archive
