# Supplementary material for: An integrated study of Violae Herba (Viola philippica) and five adulterants by morphology, chemical compositions and chloroplast genomes: insights into its certified plant origin
Source: Chin Med. 2022 Mar 3;17:32. doi: 10.1186/s13020-022-00585-9 (PMC8892722; doi:10.1186/s13020-022-00585-9)
Supplement: Supplementary file 1 — Additional file 1: Table S1. Information of 18 batches of commercial samples and tentative identification. [file 13020_2022_585_MOESM1_ESM.docx]

| **Sample** | **TCM market** | ***rbcL*a Accession Number** | ***psbA-trnH* Accession Number** | **Morphological identification** |
| --- | --- | --- | --- | --- |
| M1 | Anguo City, Hebei Province | OL441120 | - | *Viola prionantha* |
| M2 | Bozhou City, Anhui Province | OL441121 | OL441136 | *Viola philippica* and *Viola prionantha* |
| M3 | Bozhou City, Anhui Province | OL441122 | - | *Viola prionantha* and *Viola inconspicua* |
| M4 | Bozhou City, Anhui Province | OL441123 | - | Failed to identify |
| M5 | Bozhou City, Anhui Province | - | - | Failed to identify |
| M6 | Hebi City, Henan Province | OL441124 | OL441138 | *Viola prionantha* |
| M7 | Bozhou City, Anhui Province | OL441125 | OL441139 | *Viola prionantha* |
| M8 | Bozhou City, Anhui Province | OL441126 | OL441140 | *Viola philippica* or *Viola betonicifolia* |
| M9 | Suqian City, Jiangsu Province | OL441127 | OL441141 | *Viola prionantha* and *Viola inconspicua* |
| M10 | Qichun County, Hubei Province | - | - | Failed to identify |
| M11 | Shangluo City, Shaanxi Province | OL441128 | OL441142 | *Viola prionantha* and *Viola inconspicua* |
| M12 | Meizhou City, Guangdong Province | OL441129 | - | *Viola prionantha* and *Viola philippica* |
| M13 | Nanyang City, Henan Province | OL441130 | OL441143 | *Viola philippica* and *Viola prionantha* |
| M14 | Bozhou City, Anhui Province | OL441131 | - | *Viola philippica* |
| M15 | Meishan City, Sichuan Province | OL441132 | - | *Viola prionantha* |
| M16 | Jieyang City, Guangdong Province | - | - | *Viola prionantha* and *Viola inconspicua* |
| M17 | Anguo City, Hebei Province | OL441133 | OL441145 | *Viola prionantha* |
| M18 | Heze City, Shandong Province | OL441134 | OL441146 | *Viola prionantha* |

**Additional file 1: Table S1. Information of 18 batches of commercial samples and tentative identification.**
