# Supplementary material for: An integrated study of Violae Herba (Viola philippica) and five adulterants by morphology, chemical compositions and chloroplast genomes: insights into its certified plant origin
Source: Chin Med. 2022 Mar 3;17:32. doi: 10.1186/s13020-022-00585-9 (PMC8892722; doi:10.1186/s13020-022-00585-9)
Supplement: Supplementary file 3 — Additional file 3: Table S3. Sample information of six Viola species for cp genome sequencing in this study. [file 13020_2022_585_MOESM3_ESM.docx]

| **Species** | **Common synonyms** | **Chinese Name** | **Accession No.** | **Locality** | **Longitude** | **Latitude** | **Voucher No.** | **Raw Reads** | **Mapping Reads** | **Mapping Ratio** | **Biosample Accession** |
| --- | --- | --- | --- | --- | --- | --- | --- | --- | --- | --- | --- |
| *Viola inconspicua* Blume | - | Chang E Jin Cai | MZ065354 | Jiaxing City, Zhejiang, China | 120.85 | 30.70 | YLTCEJC200315JX | 36297544 | 2426398 | 6.68% | [SAMN15069690](https://dataview.ncbi.nlm.nih.gov/object/SAMN15069690) |
| *Viola betonicifolia* Sm. | *Viola betonicifolia* subsp. *nepalensis* (Ging.) W. Becker | Ji Ye Jin Cai | MT702388 | Jiaxing City, Zhejiang, China | 120.85 | 30.70 | YLTJYJC200315JX | 33220638 | 1308388 | 3.94% | [SAMN15069687](https://dataview.ncbi.nlm.nih.gov/object/SAMN15069687) |
| *Viola japonica* Langsd. ex Ging. | *Viola concordifolia* C. J. Wang | Li Tou Cao | MZ151699 | Nanjing City, Jiangsu, China | 118.83 | 32.06 | YLTLTC200310NJ | 40587526 | 2719204 | 6.70% | [SAMN15069689](https://dataview.ncbi.nlm.nih.gov/object/SAMN15069689) |
| *Viola collina* Besser | - | Qiu Guo Jin Cai | MZ169541 | Nanjing City, Jiangsu, China | 118.83 | 32.06 | YLTQGJC200310NJ | 36817238 | 3181741 | 8.64% | [SAMN15069688](https://dataview.ncbi.nlm.nih.gov/object/SAMN15069688) |
| *Viola philippica* Cav. | *Viola yedoensis* Makino | Zi Hua Di Ding | MZ221749 | Jiaxing City, Zhejiang, China | 120.85 | 30.70 | YLTZHDD200315JX | 32633358 | 3297654 | 10.11% | [SAMN15069691](https://dataview.ncbi.nlm.nih.gov/object/SAMN15069691) |
| *Viola prionantha* Bunge | - | Zao Kai Jin Cai | MZ221750 | Shijiazhuang City, Hebei, China | 114.52 | 38.05 | YLTZKJC200322HB | 53658183 | 4874163 | 9.08% | [SAMN15069692](https://dataview.ncbi.nlm.nih.gov/object/SAMN15069692) |

**Additional file 3: Table S3. Sample information of six *Viola* species for cp genome sequencing in this study.**
