# Supplementary material for: An integrated study of Violae Herba (Viola philippica) and five adulterants by morphology, chemical compositions and chloroplast genomes: insights into its certified plant origin
Source: Chin Med. 2022 Mar 3;17:32. doi: 10.1186/s13020-022-00585-9 (PMC8892722; doi:10.1186/s13020-022-00585-9)
Supplement: Supplementary file 4 — Additional file 4: Figure S1. The distribution schematic of the six Viola species. Figure S2. Leaf morphology of the 18 commercial VH for tentative identification. Figure S3. The HPLC chromatograms of the wild-collected samples of 6 Viola species. Figure S4. The total ion current chromatograms of six Viola species in negative and positive ion mode. Figure S5. The distribution of RNA editing sites. Figure S6. Codon numbers for each amino acid of the six Viola species. [file 13020_2022_585_MOESM4_ESM.docx]

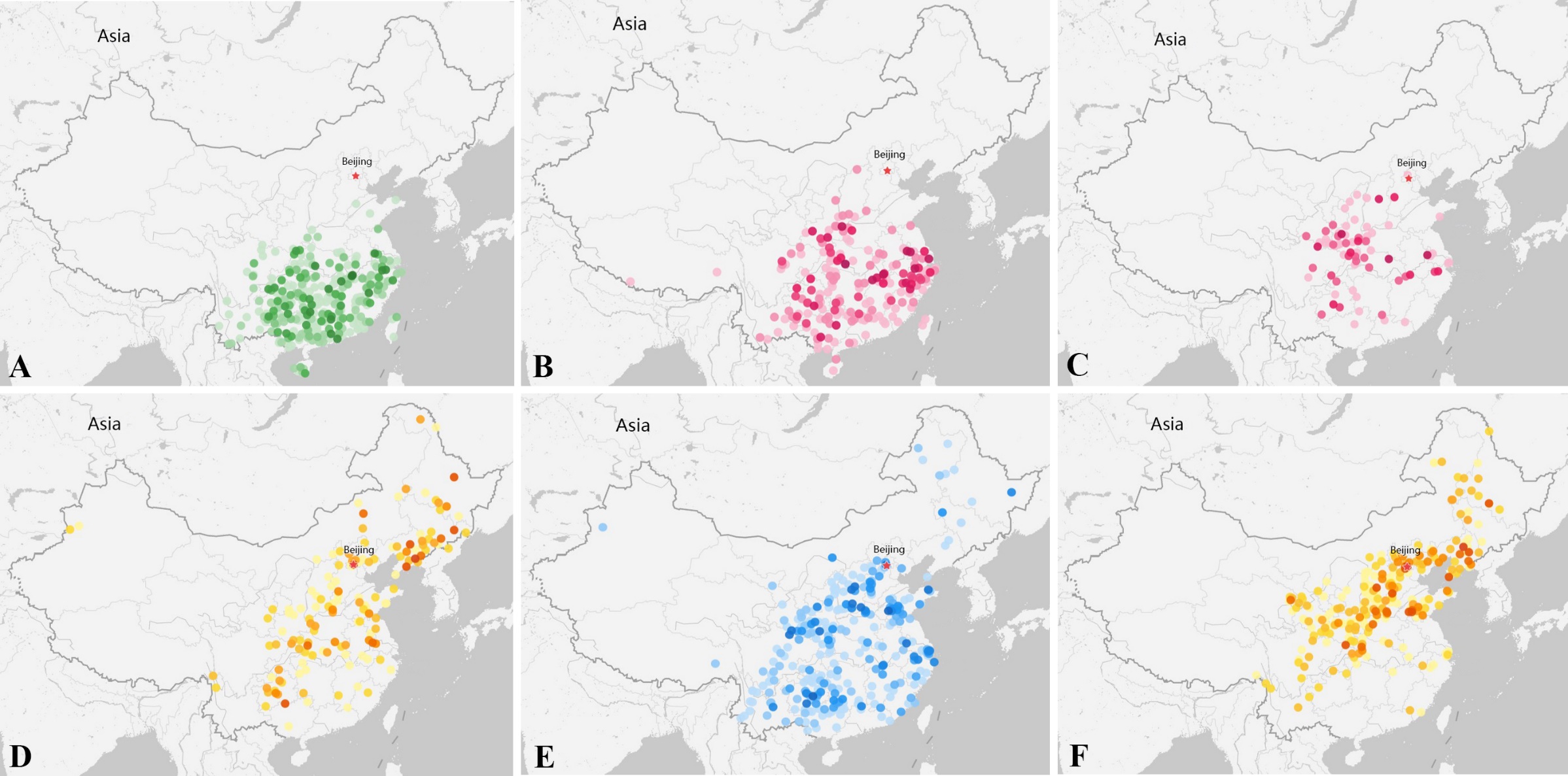


**Figure S1.** The distribution schematic of the six *Viola* species. **A**: *V. inconspicua*; **B**: *V. betonicifolia*; **C**: *V. japonica*; **D**: *V. collina*; **E**: *V. philippica*; **F**: *V. prionantha*.


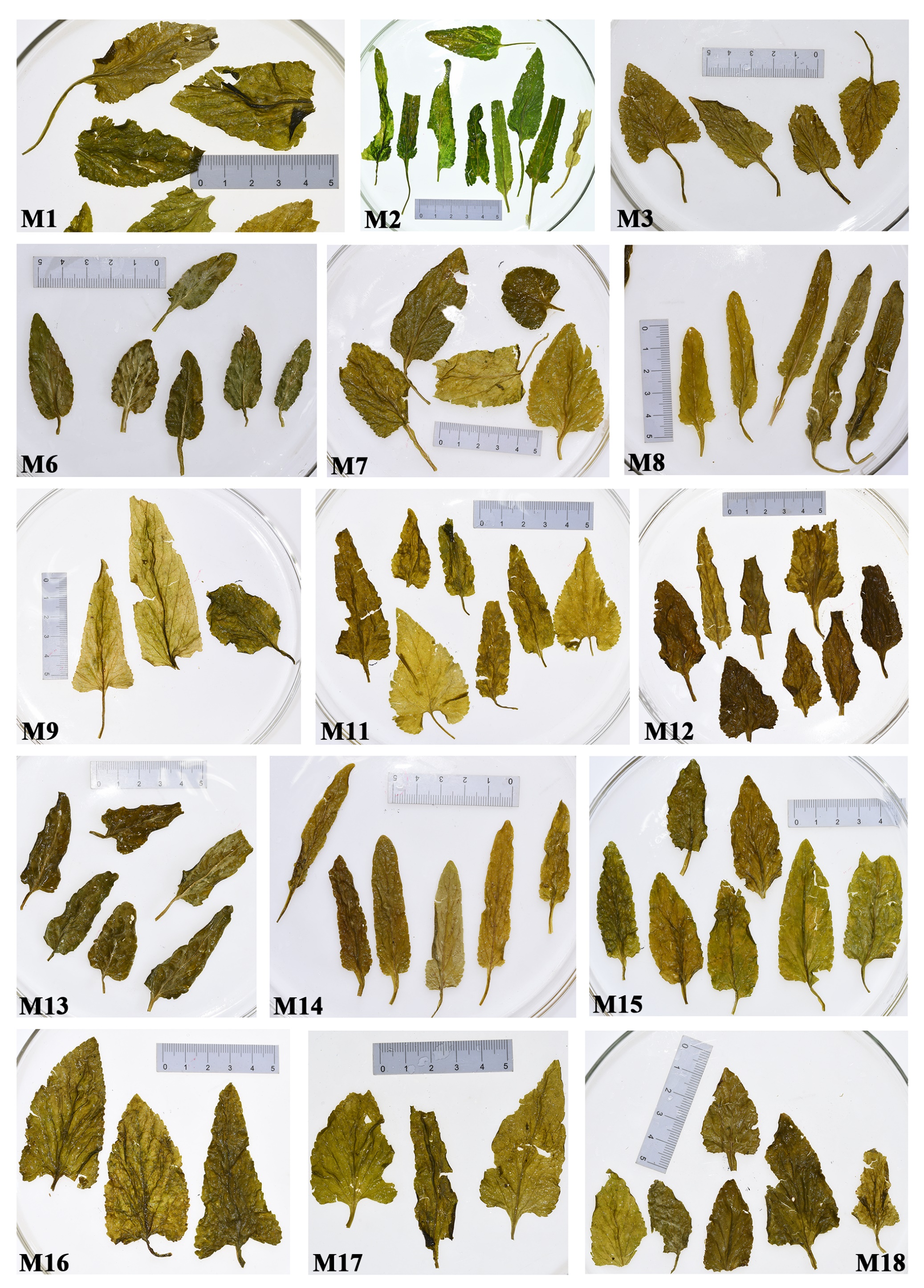


**Figure S2**. Leaf morphology of the 18 commercial VH for tentative identification.


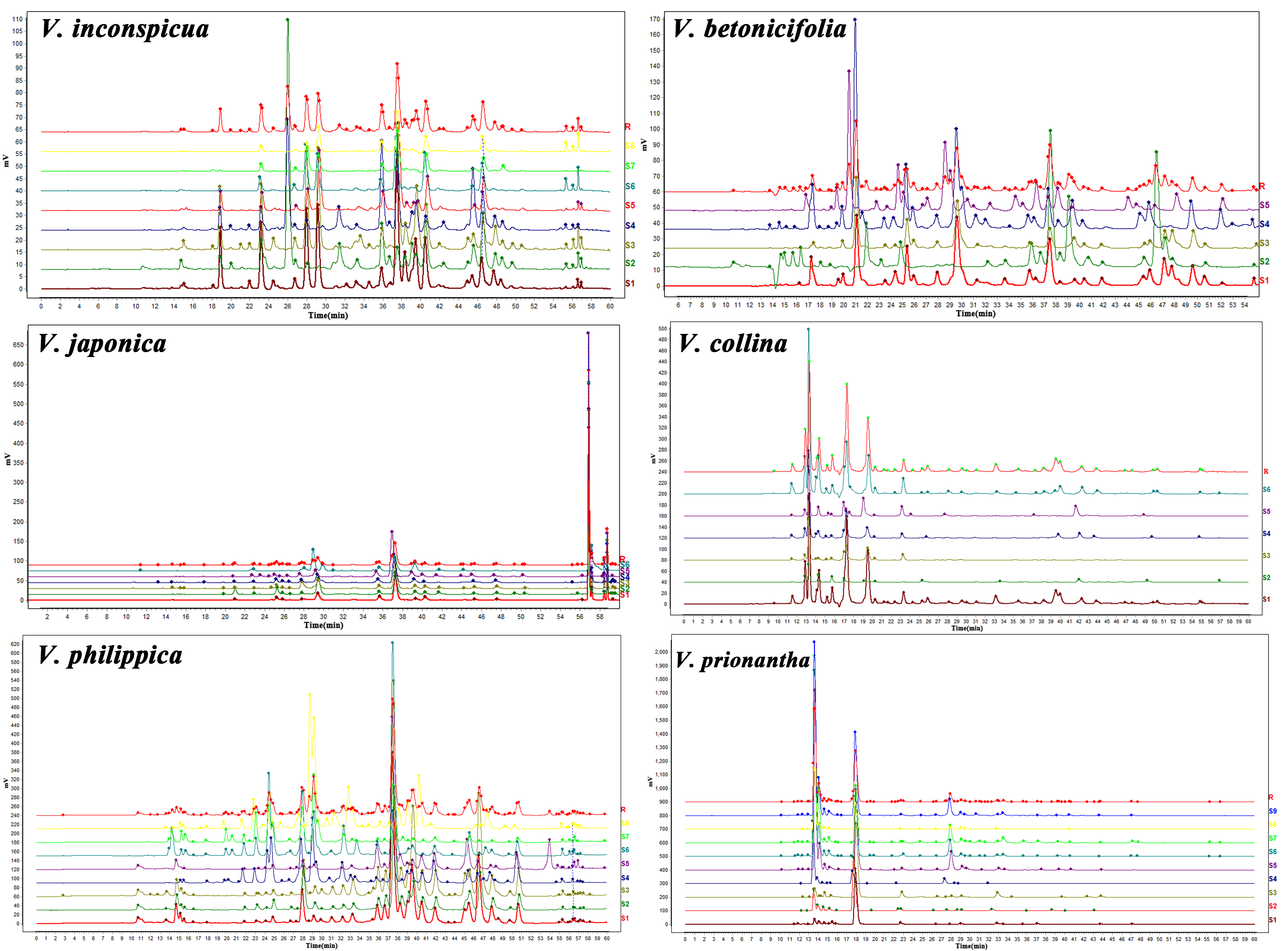


**Figure S3**. The HPLC chromatograms of the wild-collected samples of 6 *Viola* species. The reference characteristic spectrum of each species labeled as “R” is generated for the following comparative analysis.

**
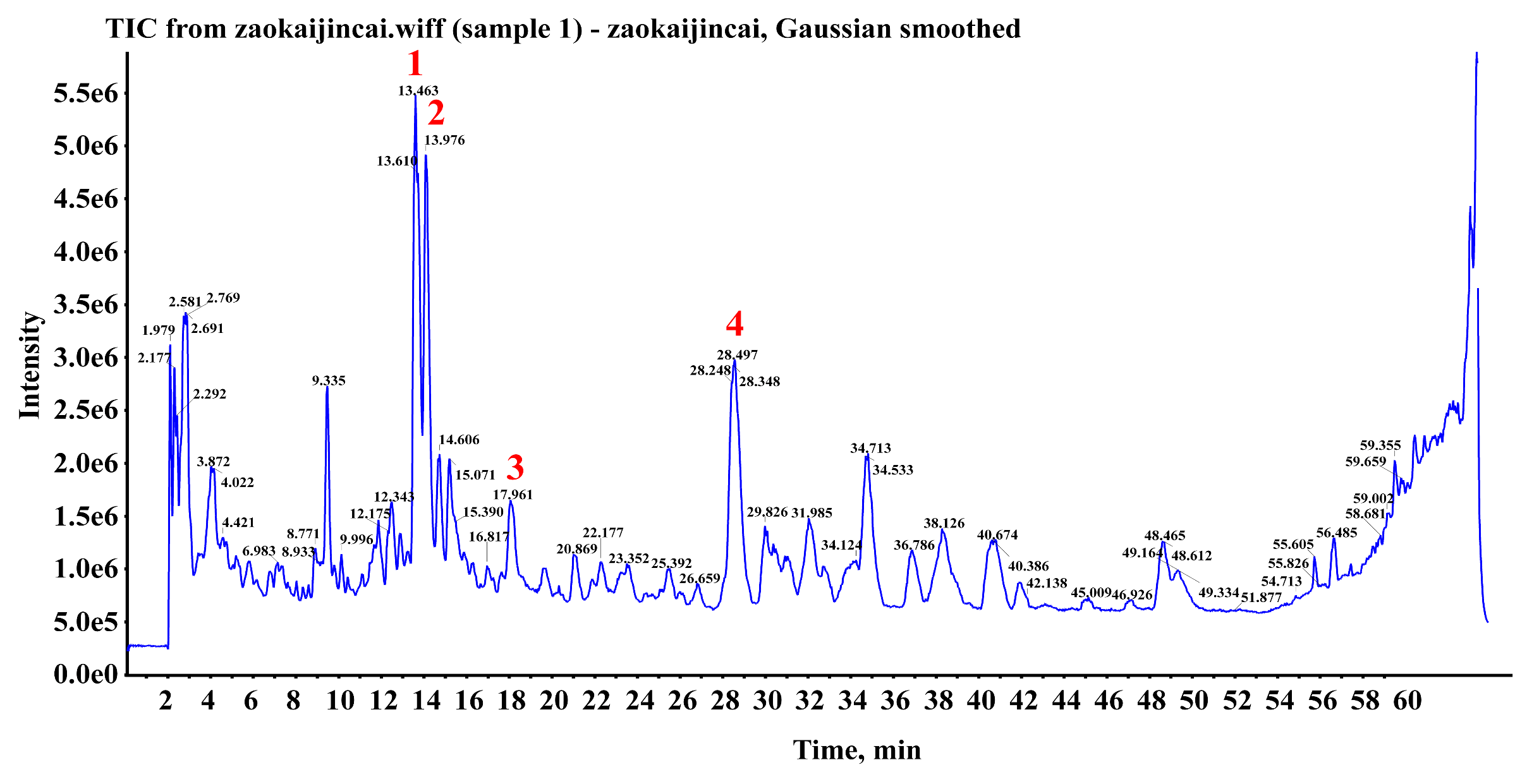
**

**A**

**B**

**
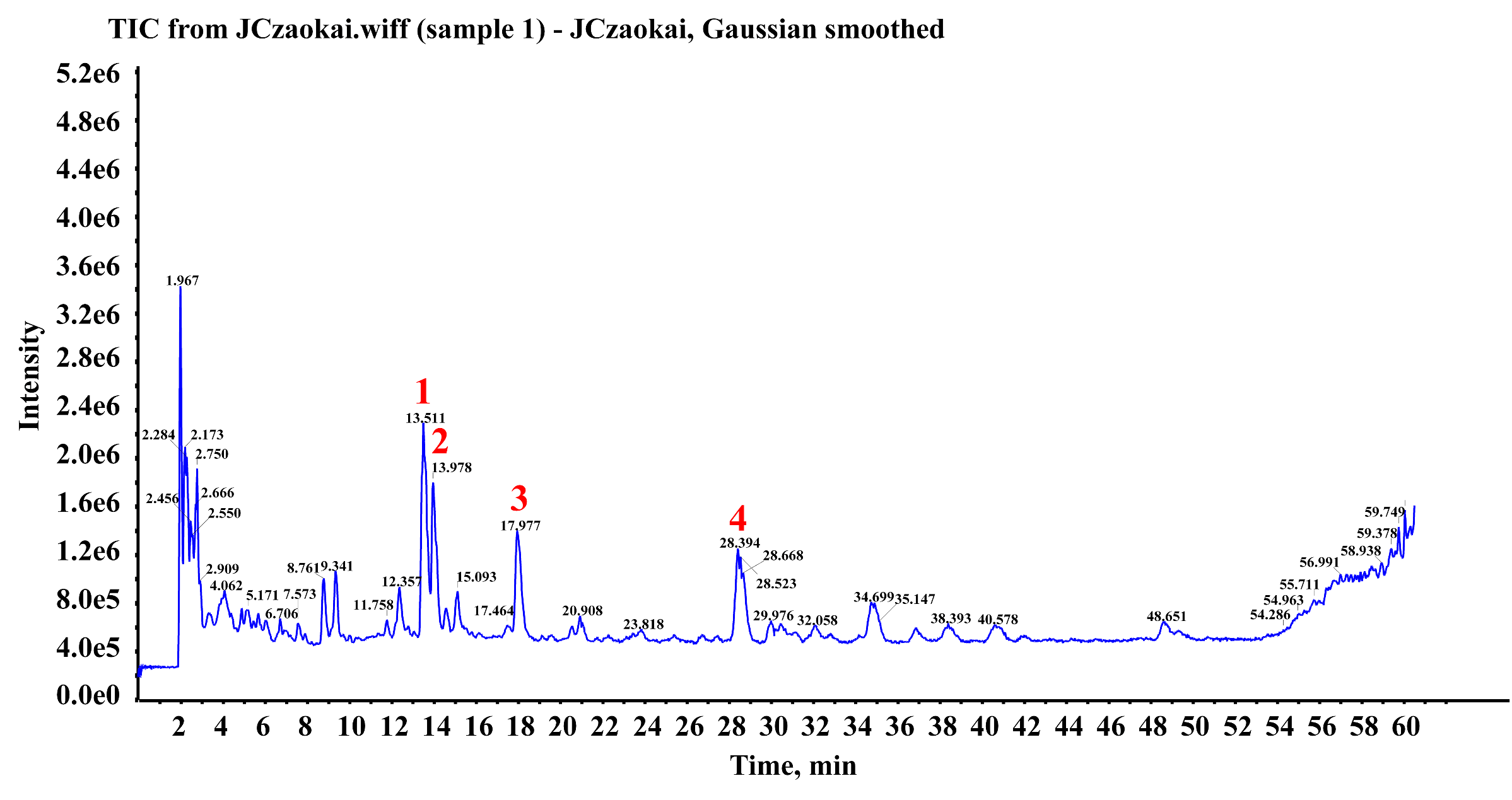
**

**Figure S4-1**. The total ion current chromatograms of *Viola prionantha* in negative (**A**) and positive (**B**) ion mode. The numbers 1 to 4 indicating cichoriin, esculin, esculetin, and prionanthoside respectively.


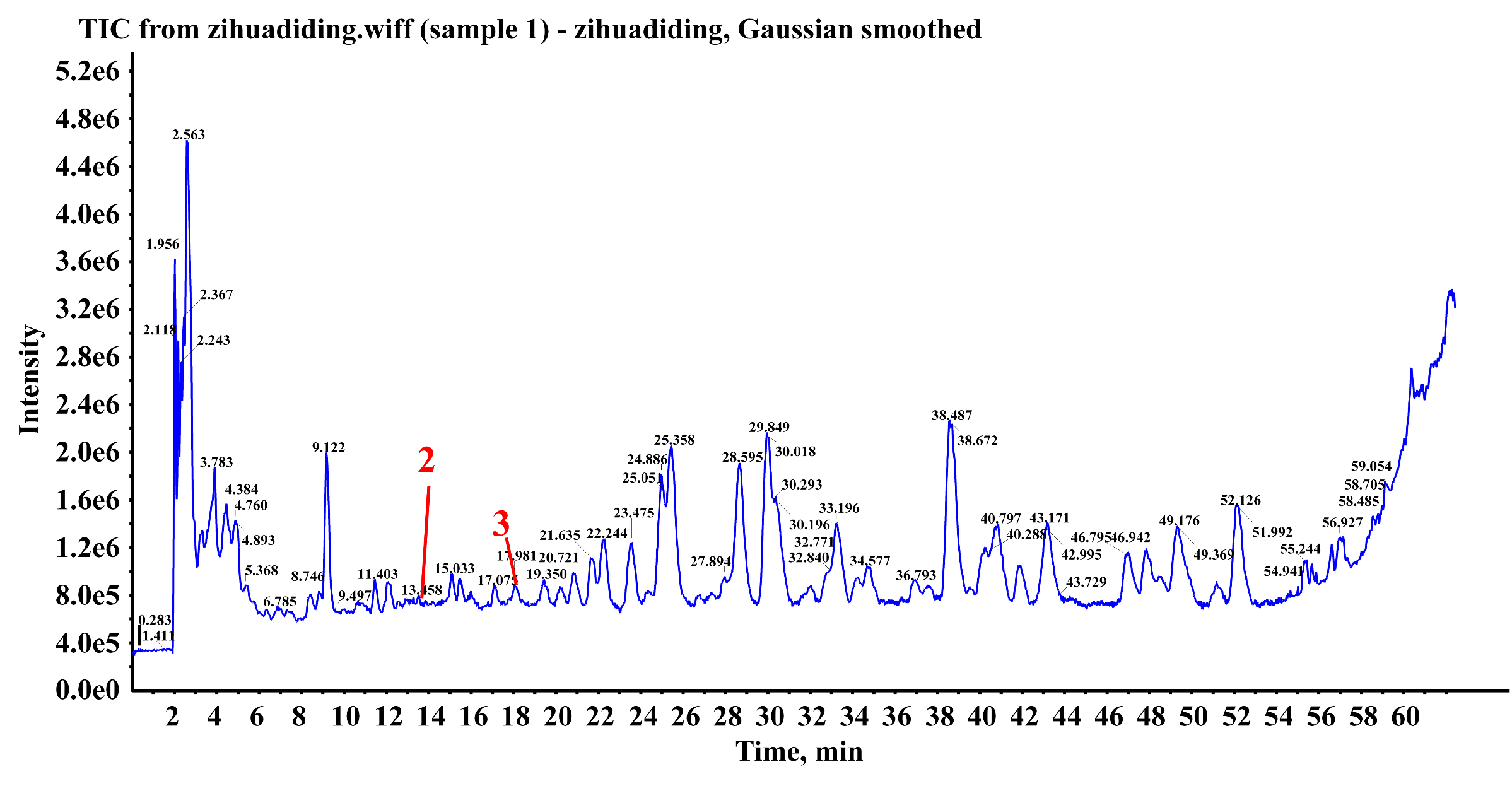


**A**

**B**


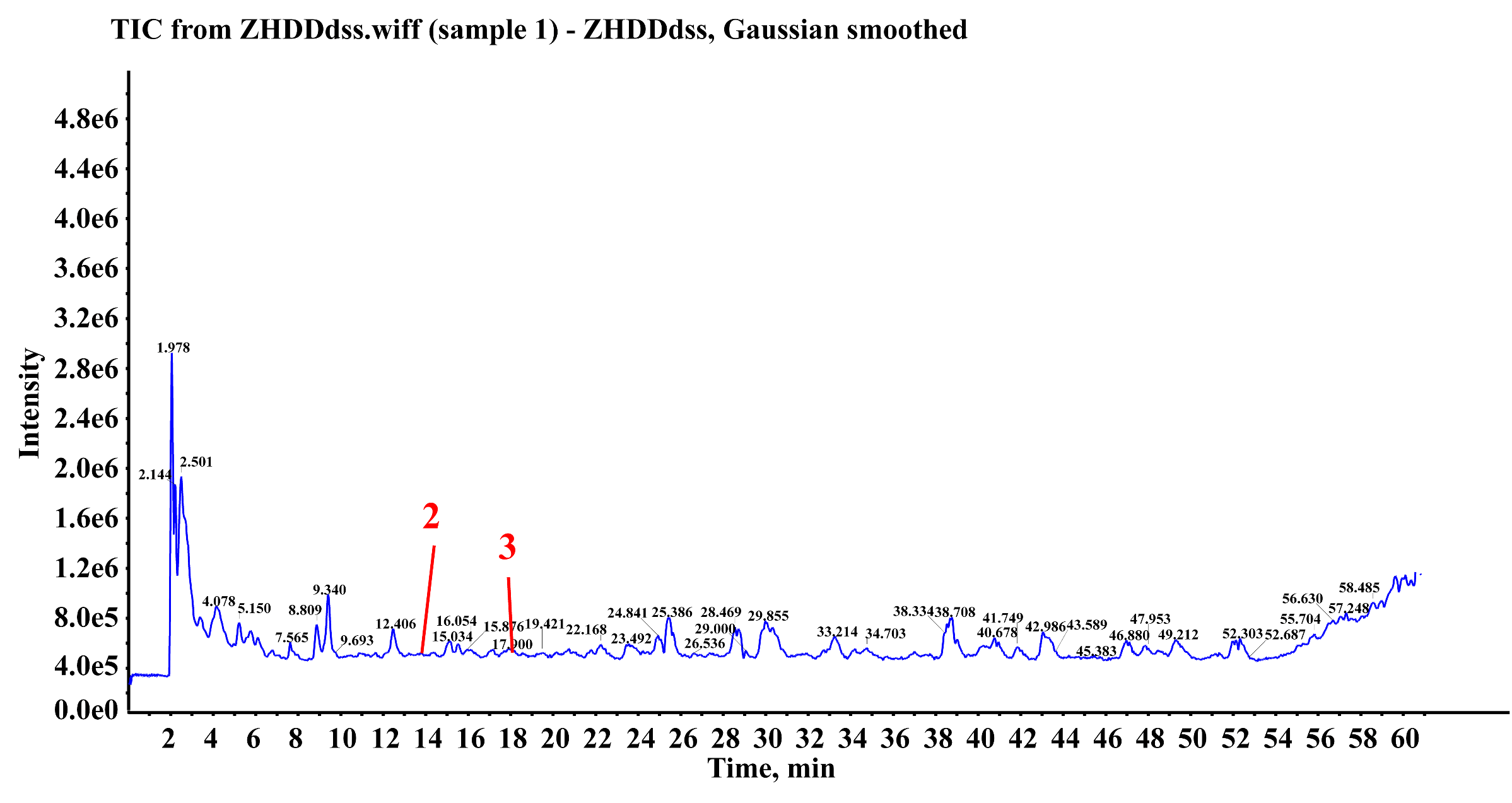


**Figure S4-2**. The total ion current chromatograms of *Viola philippica* in negative (**A**) and positive (**B**) ion mode. The numbers 2 and 3 indicating esculin and esculetin respectively.


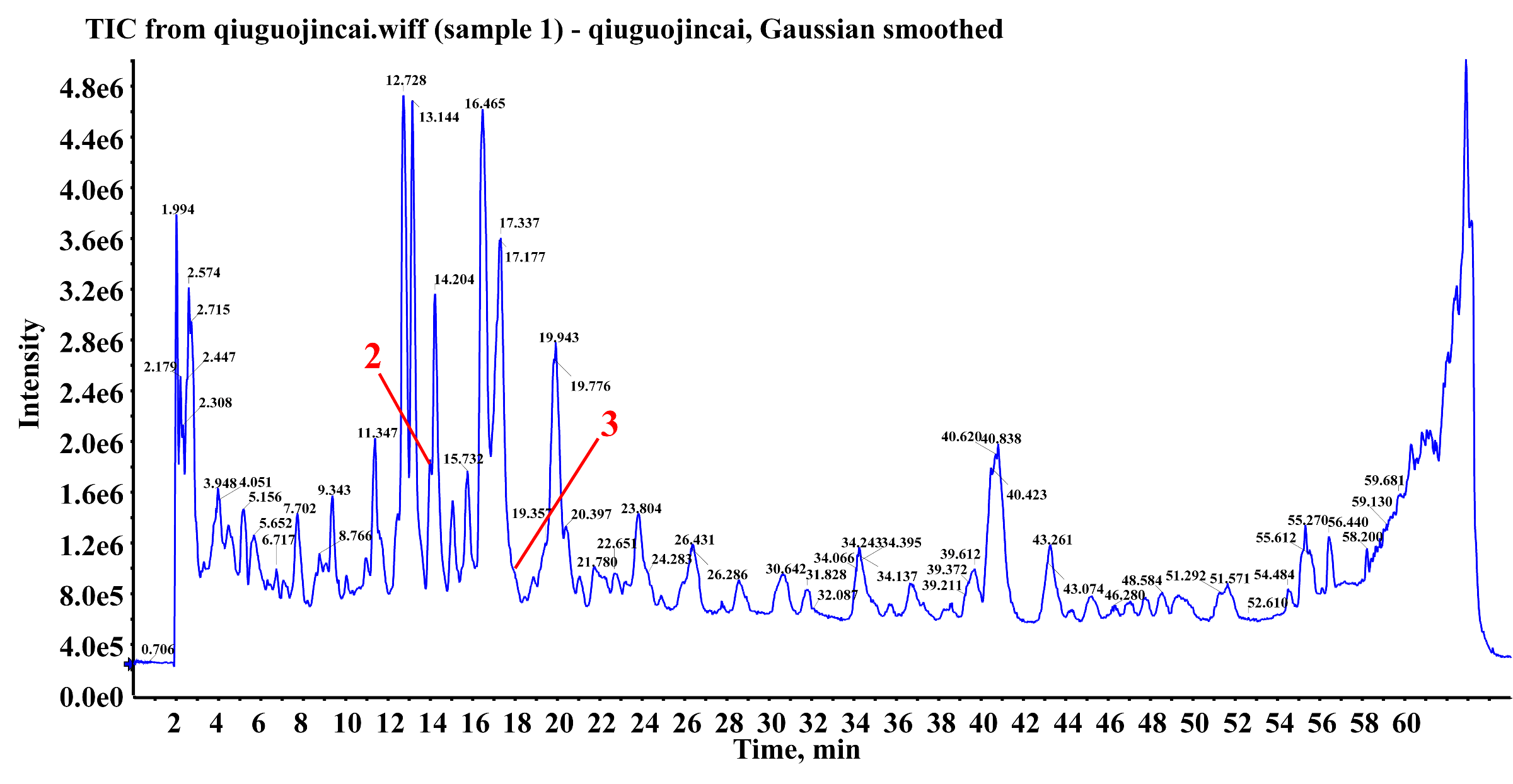


**A**

**B**


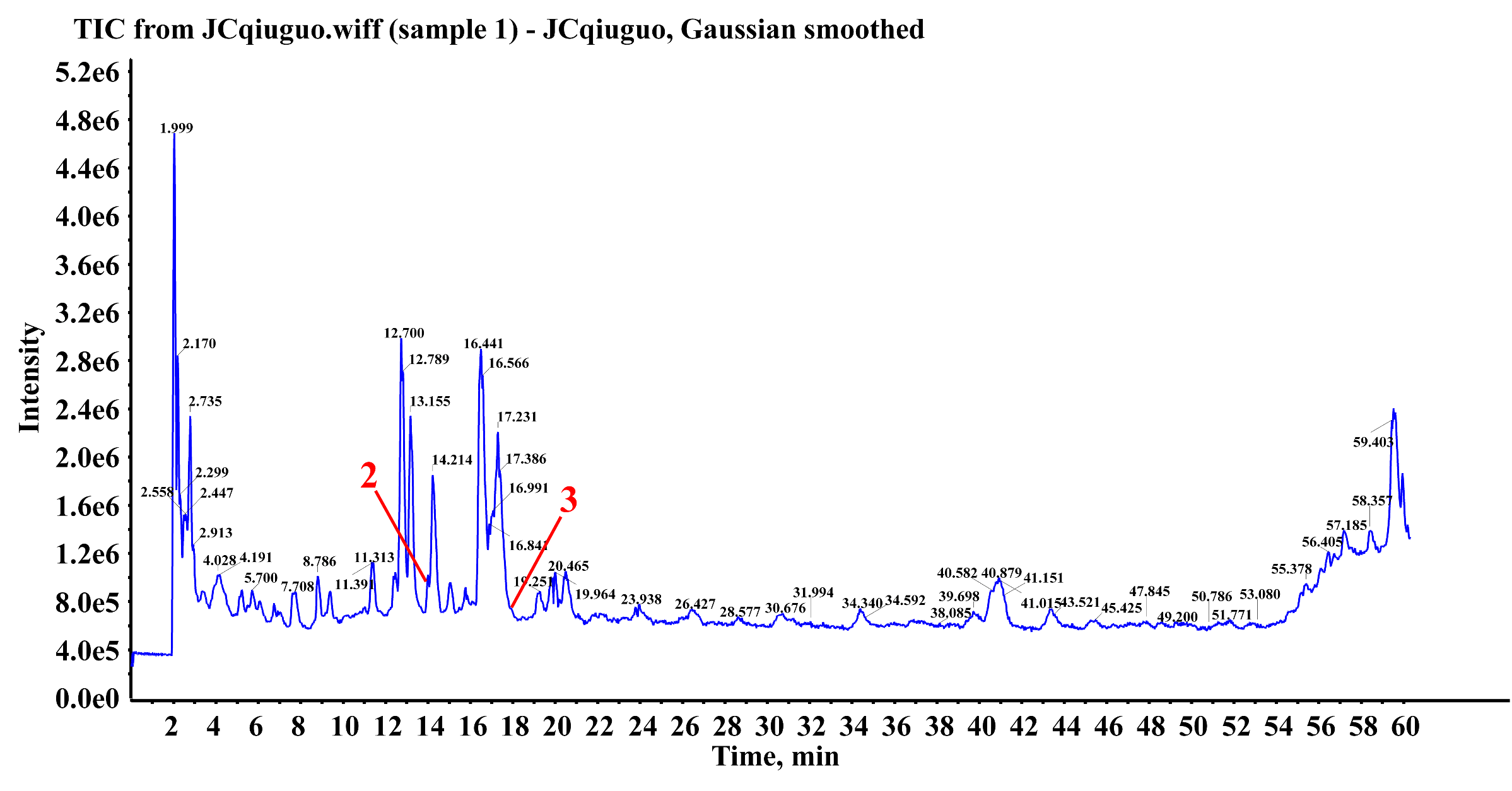


**Figure S4-3**. The total ion current chromatograms of *Viola collina* in negative (**A**) and positive (**B**) ion mode. The numbers 2 and 3 indicating esculin and esculetin respectively.


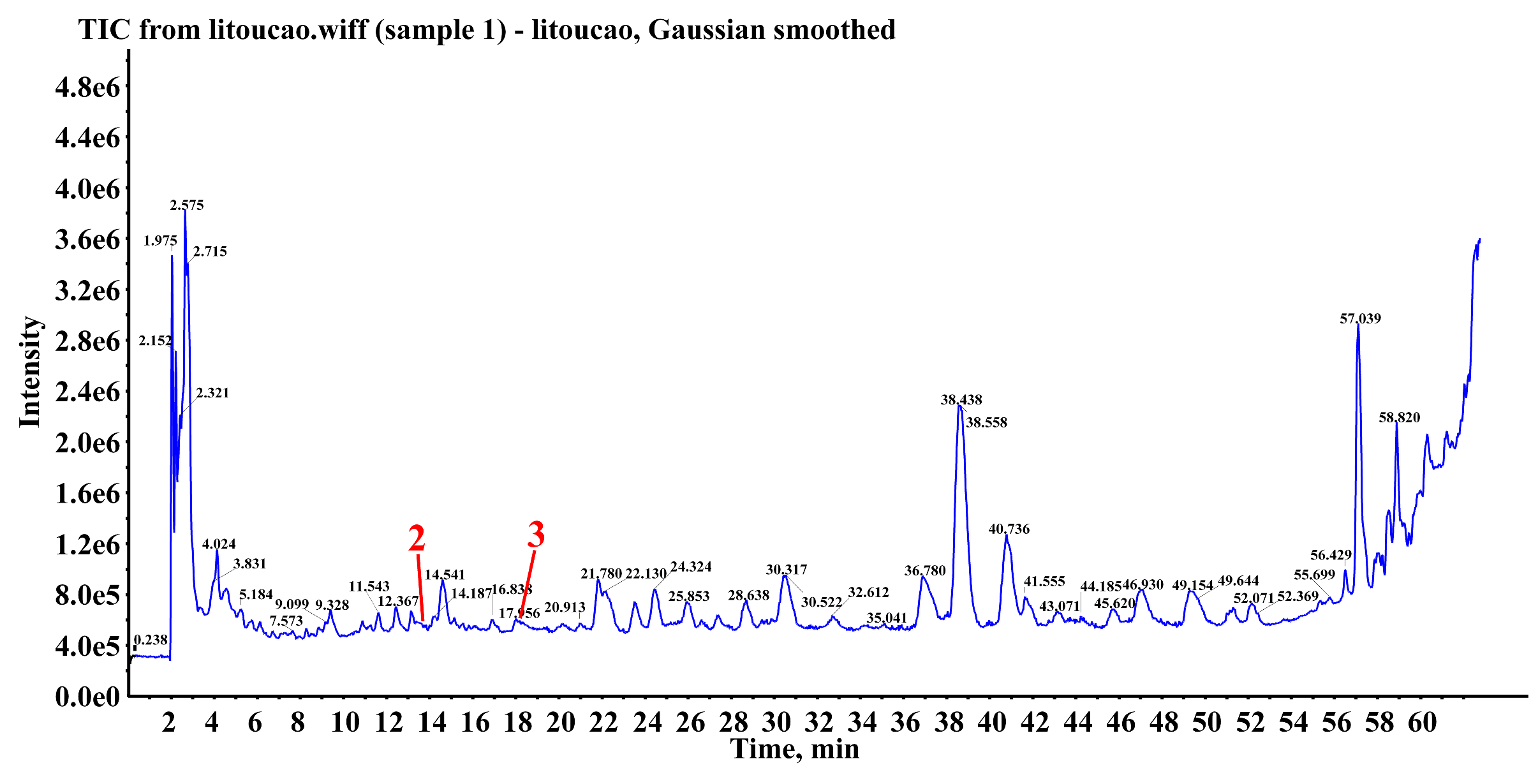


**A**

**B**


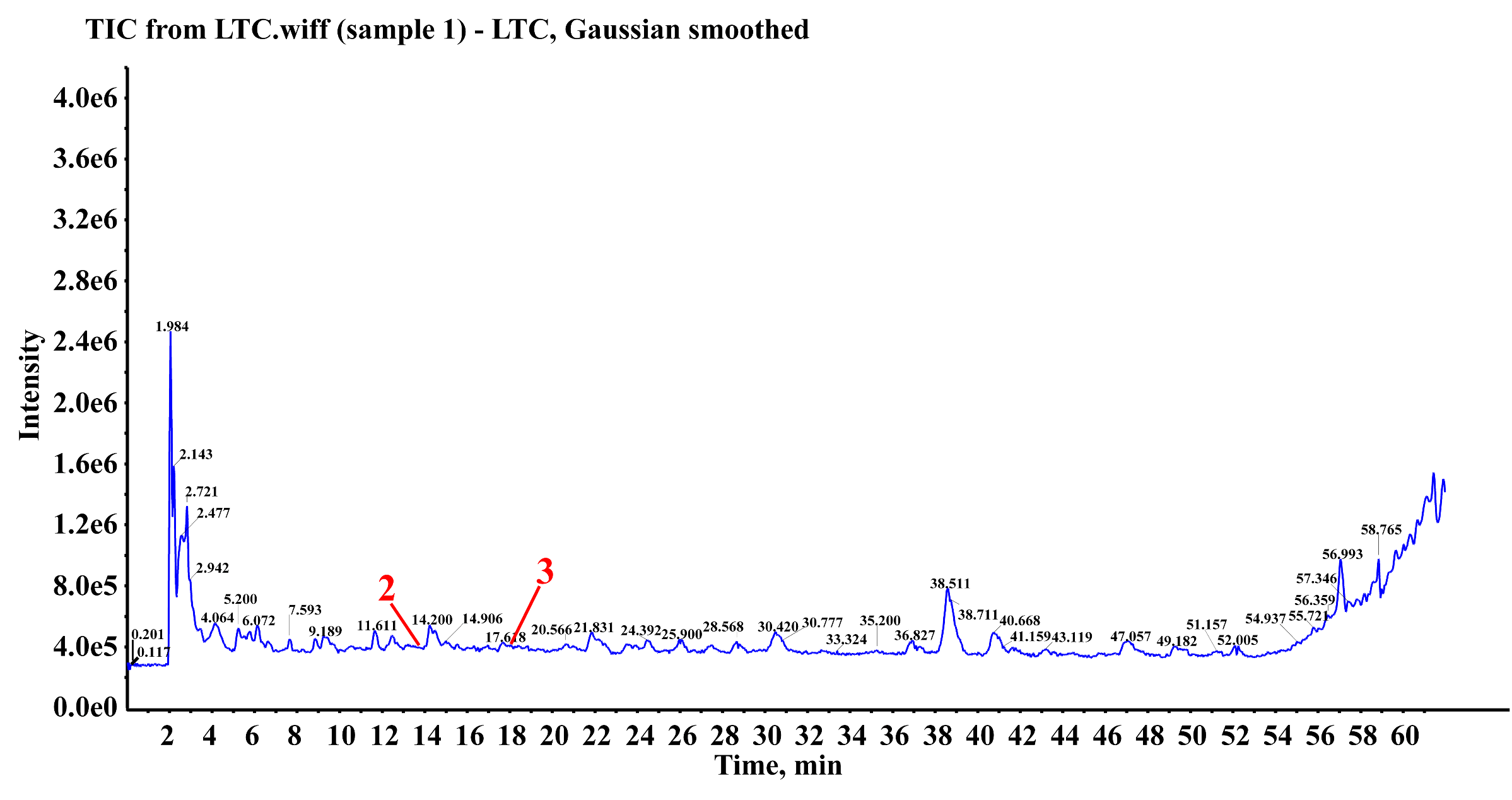


**Figure S4-4**. The total ion current chromatograms of *Viola japonica* in negative (**A**) and positive (**B**) ion mode. The numbers 2 and 3 indicating esculin and esculetin respectively.


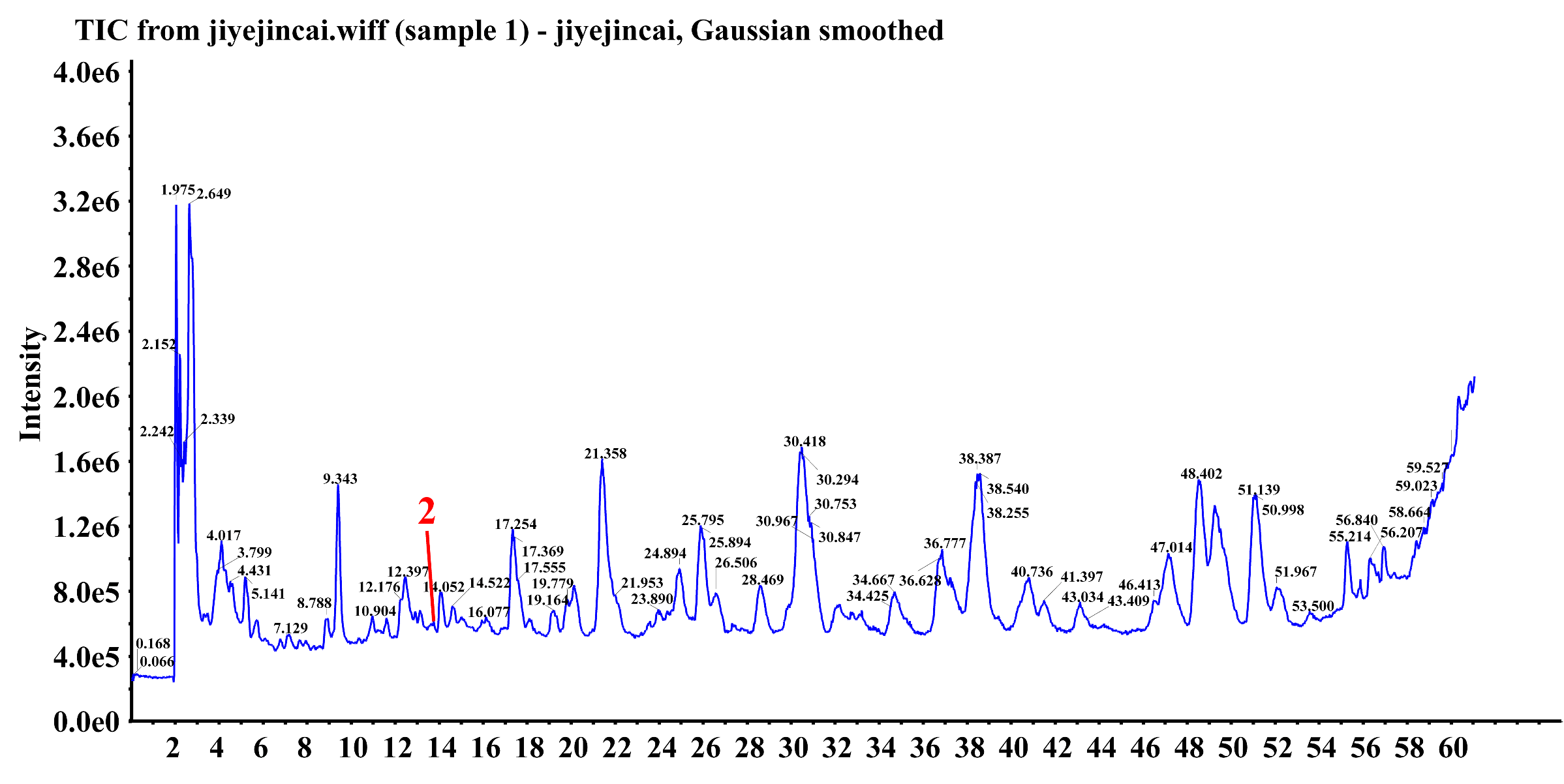


**B**

**A**


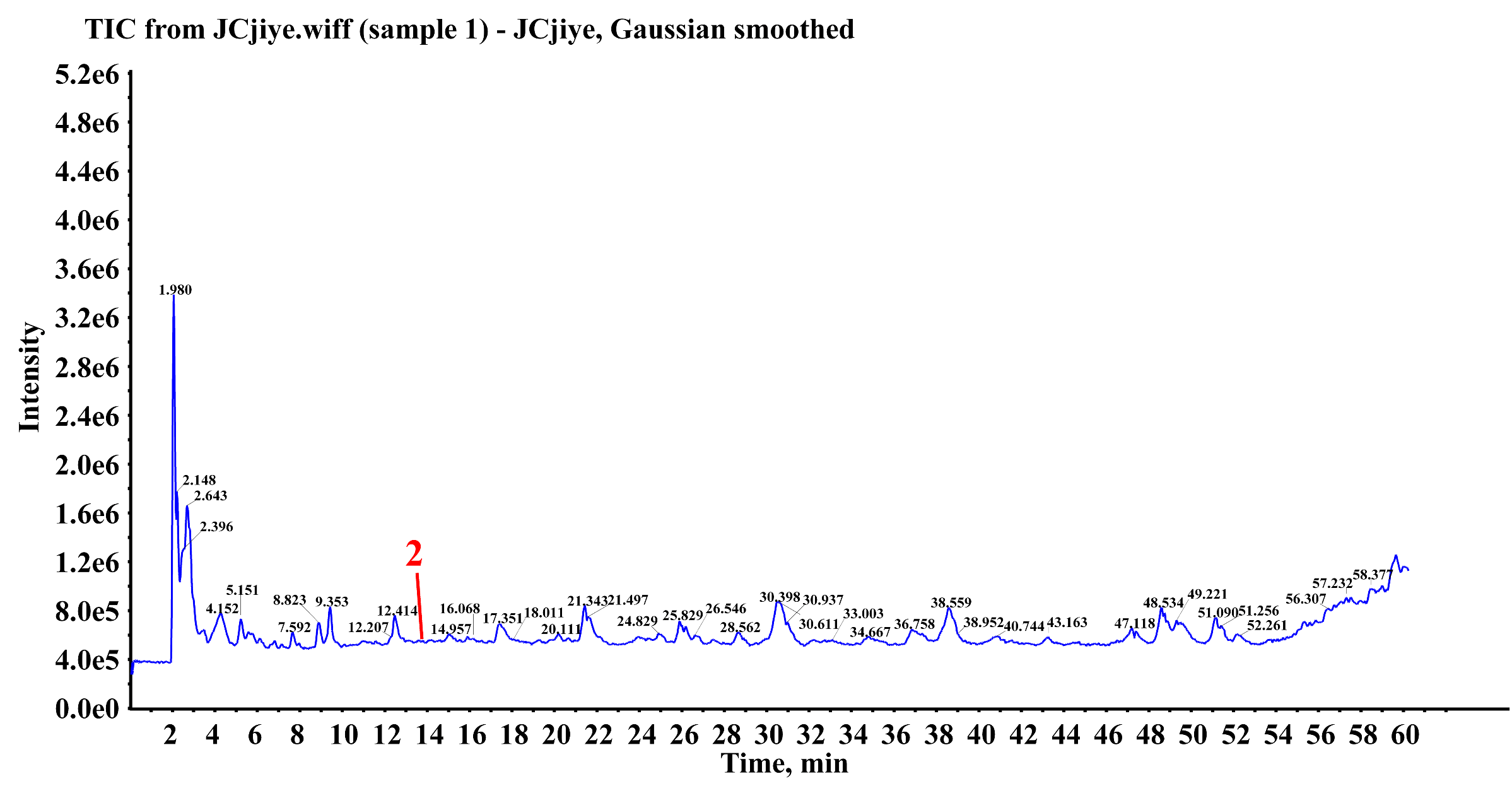


**Figure S4-5**. The total ion current chromatograms of *Viola betonicifolia* in negative (**A**) and positive (**B**) ion mode. The number 2 indicating esculin.


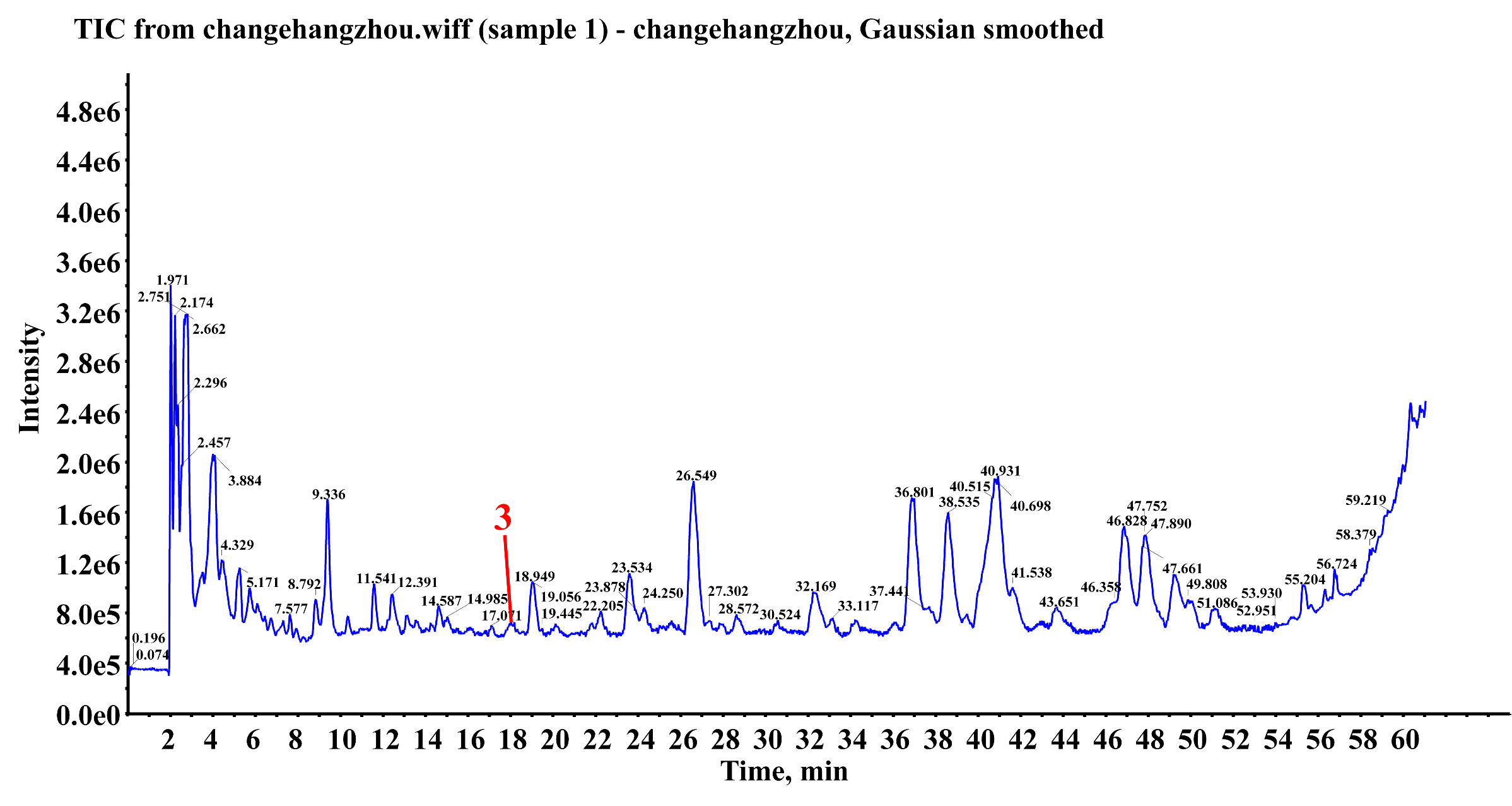


**A**

**B**


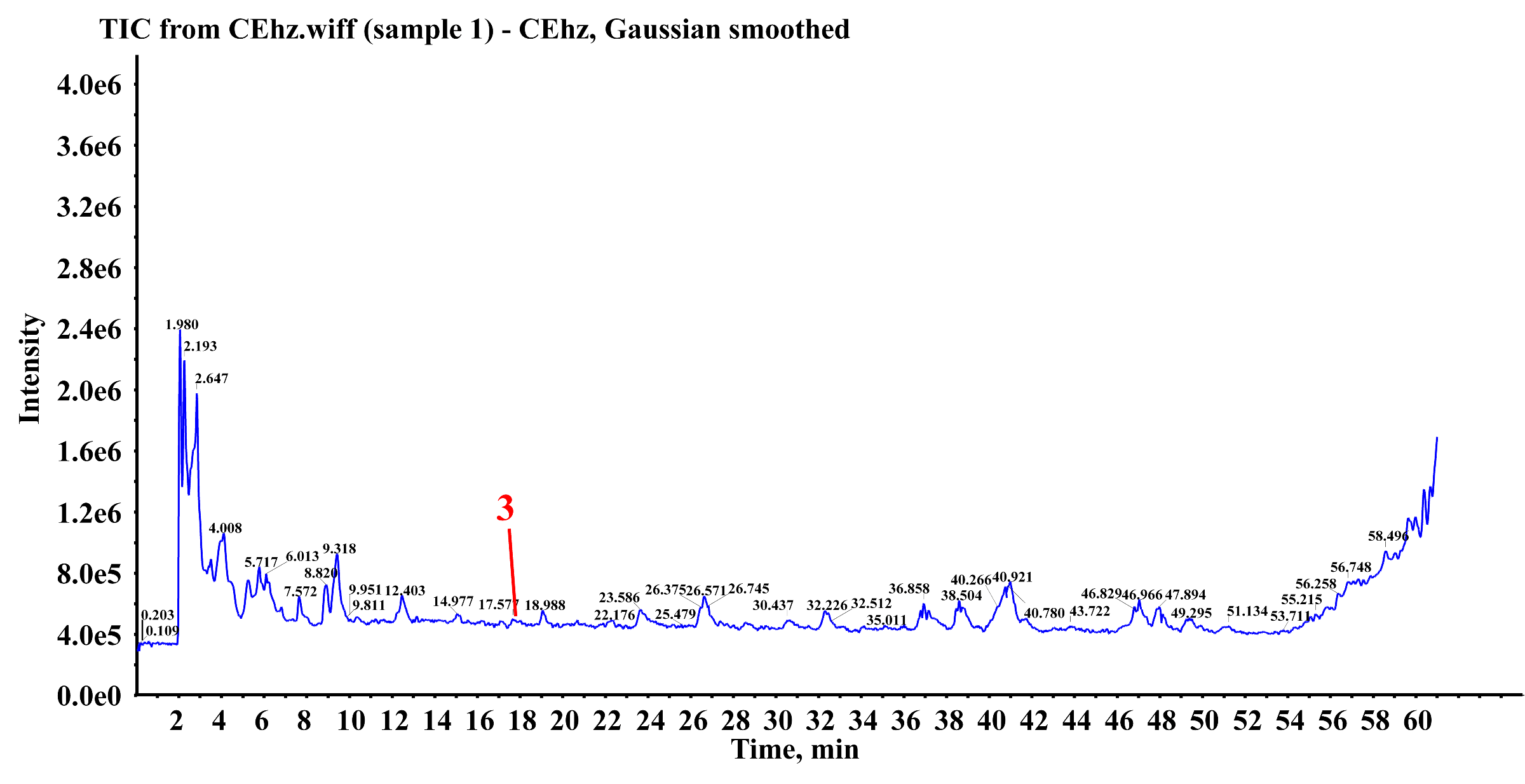


**Figure S4-6**. The total ion current chromatograms of *Viola inconspicua* in negative (**A**) and positive (**B**) ion mode. The number 3 indicating esculetin.


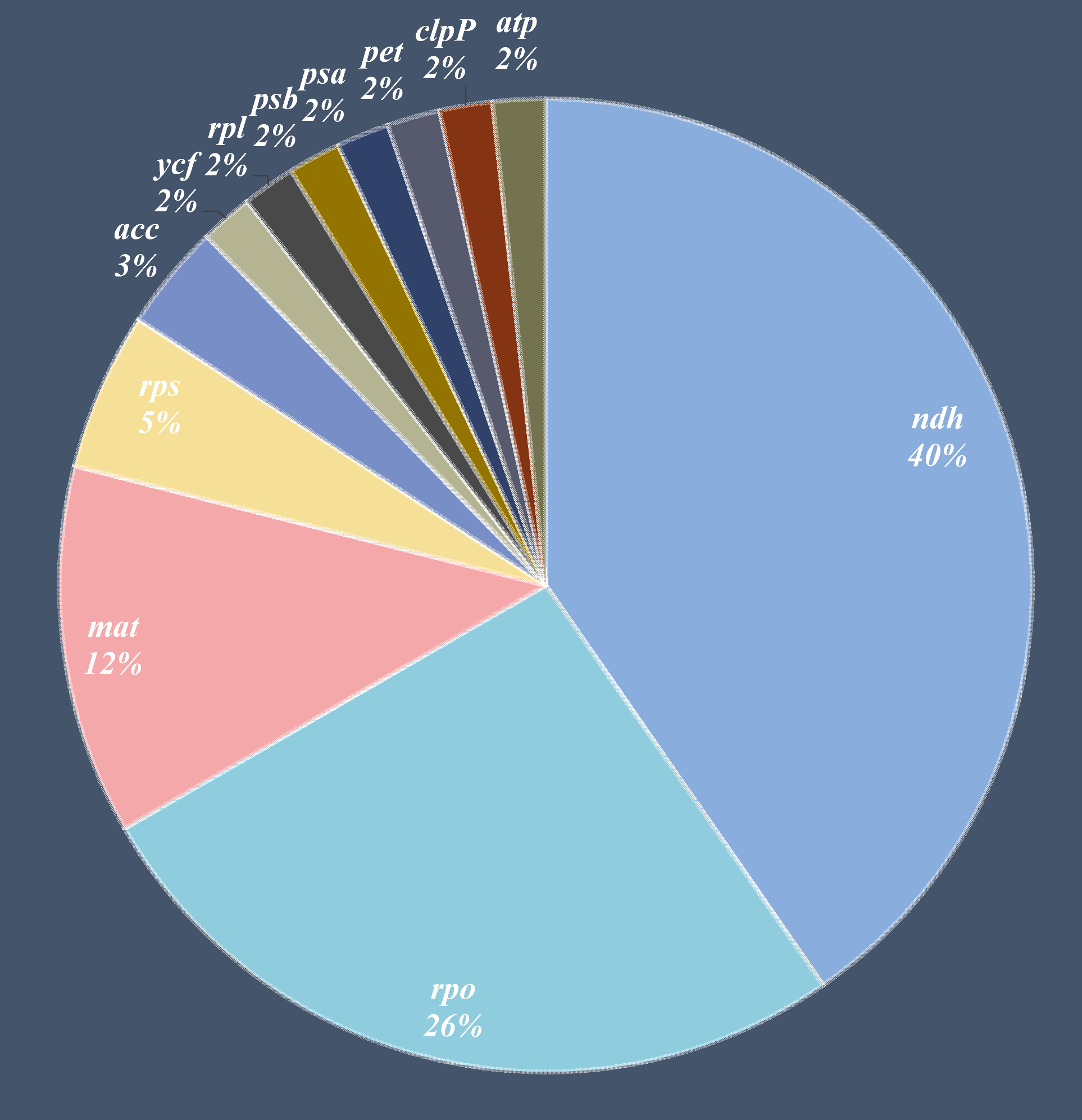


**Figure S5**. The distribution of RNA editing sites.


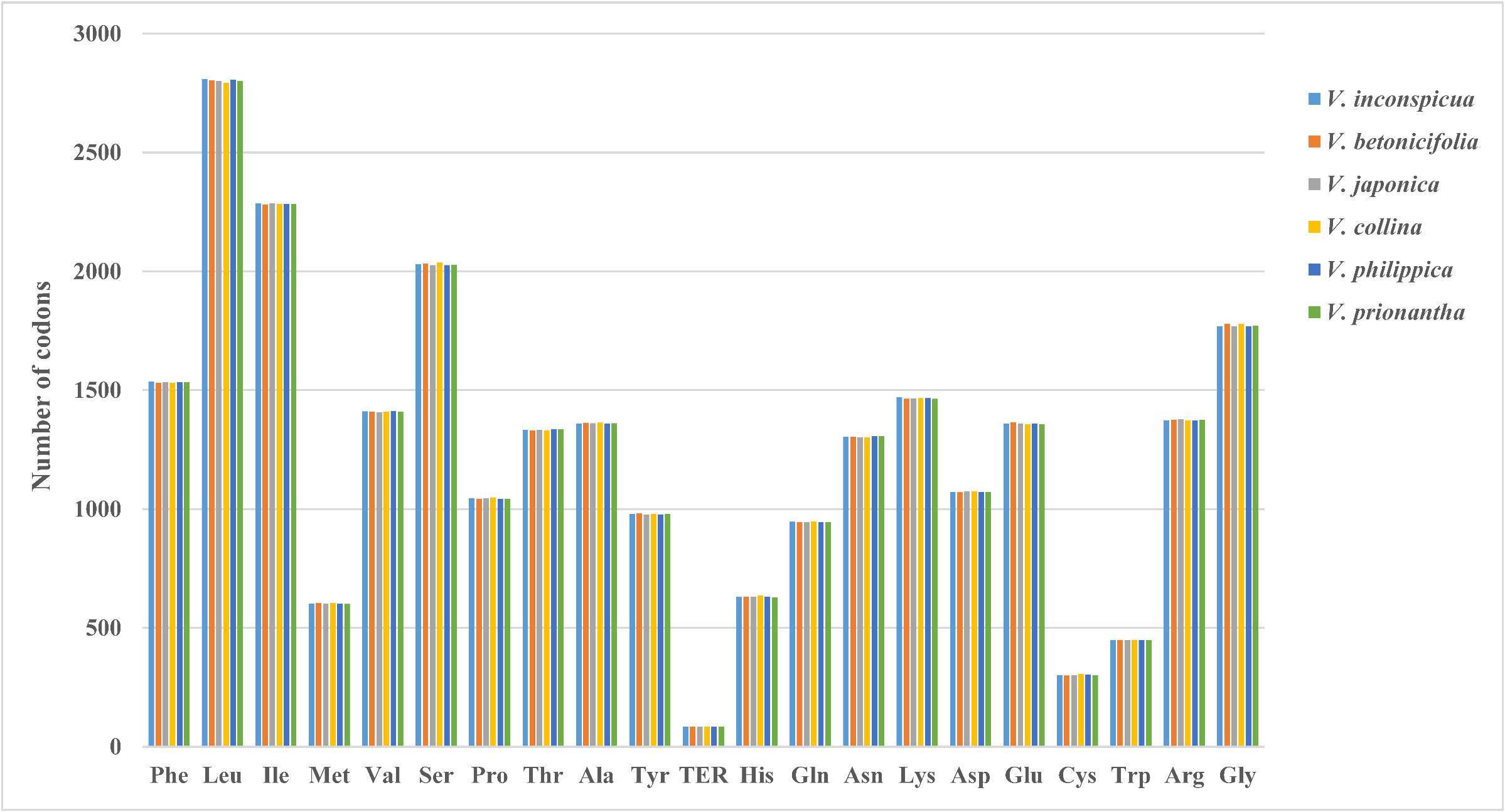


**Figure S6**. Codon numbers for each amino acid of the six *Viola* species.
