# Supplementary material for: An integrated study of Violae Herba (Viola philippica) and five adulterants by morphology, chemical compositions and chloroplast genomes: insights into its certified plant origin
Source: Chin Med. 2022 Mar 3;17:32. doi: 10.1186/s13020-022-00585-9 (PMC8892722; doi:10.1186/s13020-022-00585-9)
Supplement: Supplementary file 5 — Additional file 5: Table S4. Main chemical components determined by HPLC-Triple-TOF-MS/MS. [file 13020_2022_585_MOESM5_ESM.docx]

**Additional file 5: Table S4. Main chemical components determined by HPLC-Triple-TOF-MS/MS.**

| **No.** | **Species** | **Rt**  **(min)** | **Formula** | **Quasi-molecular ion** | **m/z** | **Fragment ions** | **Error**  **(ppm)** | **Compound** |
| --- | --- | --- | --- | --- | --- | --- | --- | --- |
| 1 | *V. philippica* | **/** | C_15_H_16_O_9_ | **/** | **/** | **/** | **/** | Cichoriin |
|  | *V. prionantha* | 13.490 |  | [M+H]^+^ | 341.0862 | 179, 151, 133, 123 | -1.6 |  |
|  | *V. collina* | **/** |  | **/** | **/** | **/** | **/** |  |
|  | *V. japonica* | **/** |  | **/** | **/** | **/** | **/** |  |
|  | *V. betonicifolia* | **/** |  | **/** | **/** | **/** | **/** |  |
|  | *V. inconspicua* | **/** |  | **/** | **/** | **/** | **/** |  |
| 2 | *V. philippica* | 13.938 | C_15_H_16_O_9_ | [M+H]^+^ | 341.0872 | 179, 151, 133, 123 | 1.4 | Esculin |
|  | *V. prionantha* | 13.939 |  | [M-H]^-^ | 339.0723 | 177, 133, 105 | 3.6 |  |
|  | *V. collina* | 13.912 |  | [M+H]^+^ | 341.0873 | 179, 151, 133, 123 | 1.7 |  |
|  | *V. japonica* | 13.963 |  | [M+H]^+^ | 341.0868 | 179, 151, 133, 123 | 0,2 |  |
|  | *V. betonicifolia* | 13.925 |  | [M+H]^+^ | 341.0880 | 179, 151, 133, 123 | 3.8 |  |
|  | *V. inconspicua* | **/** |  | **/** | **/** | **/** | **/** |  |
| 3 | *V. philippica* | 17.961 | C_9_H_6_O_4_ | [M+H]^+^ | 179.0335 | 151, 133, 123, 105 | -2.2 | Esculetin |
|  | *V. prionantha* | 17.867 |  | [M+H]^+^ | 179.0329 | 151, 133, 123, 105 | -5.4 |  |
|  | *V. collina* | 17.909 |  | [M+H]^+^ | 179.0342 | 151, 133, 123, 105 | 1.7 |  |
|  | *V. japonica* | 17.951 |  | [M+H]^+^ | 179.0338 | 151, 133, 123, 105 | -.07 |  |
|  | *V. betonicifolia* | **/** |  | **/** | **/** | **/** | **/** |  |
|  | *V. inconspicua* | 17.913 |  | [M+H]^+^ | 179.0344 | 151, 133, 123, 105 | 2.7 |  |
| 4 | *V. philippica* | **/** | C_17_H_18_O_10_ | **/** | **/** | **/** | **/** | Prionanthoside |
|  | *V. prionantha* | 28.282 |  | [M+H]^+^ | 383.0961 | 341, 179, 151, 133 | -3.1 |  |
|  | *V. collina* | **/** |  | **/** | **/** | **/** | **/** |  |
|  | *V. japonica* | **/** |  | **/** | **/** | **/** | **/** |  |
|  | *V. betonicifolia* | **/** |  | **/** | **/** | **/** | **/** |  |
|  | *V. inconspicua* | **/** |  | **/** | **/** | **/** | **/** |  |
