# Supplementary material for: An integrated study of Violae Herba (Viola philippica) and five adulterants by morphology, chemical compositions and chloroplast genomes: insights into its certified plant origin
Source: Chin Med. 2022 Mar 3;17:32. doi: 10.1186/s13020-022-00585-9 (PMC8892722; doi:10.1186/s13020-022-00585-9)
Supplement: Supplementary file 6 — Additional file 6: Table S5. The basic features of functional regions of cp genomes of six Viola species. [file 13020_2022_585_MOESM6_ESM.docx]

|  |  | ***V. inconspicua*** | ***V. betonicifolia*** | ***V. japonica*** | ***V. collina*** | ***V. philippica*** | ***V. prionantha*** |
| --- | --- | --- | --- | --- | --- | --- | --- |
| **Coding region** | **Protein coding region** |  |  |  |  |  |  |
|  | Length (bp) | 80662 | 80625 | 80604 | 80603 | 80599 | 80564 |
|  | GC content (%) | 37.06 | 37.09 | 37.08 | 37.17 | 37.06 | 37.06 |
|  | Length (%) | 51.25 | 51.55 | 51.20 | 50.99 | 51.20 | 51.24 |
|  | **rRNA genes** |  |  |  |  |  |  |
|  | Length (bp) | 9050 | 9050 | 9050 | 9050 | 9050 | 9050 |
|  | GC content (%) | 55.38 | 55.40 | 55.38 | 55.38 | 55.38 | 55.38 |
|  | Length (%) | 5.75 | 5.79 | 5.75 | 5.73 | 5.75 | 5.76 |
|  | **tRNA genes** |  |  |  |  |  |  |
|  | Length (bp) | 2810 | 2810 | 2810 | 2810 | 2810 | 2810 |
|  | GC content (%) | 52.99 | 52.95 | 52.99 | 52.92 | 52.99 | 52.99 |
|  | Length (%) | 1.79 | 1.80 | 1.79 | 1.78 | 1.79 | 1.79 |
| **Non-coding region** | **Intergenic Region** |  |  |  |  |  |  |
|  | Length (bp) | 46480 | 45569 | 46583 | 47239 | 46574 | 46442 |
|  | GC content (%) | 29.97 | 30.11 | 29.99 | 29.82 | 29.94 | 29.94 |
|  | Length (%) | 29.53 | 29.14 | 29.59 | 29.89 | 29.59 | 29.54 |
|  | **Intron** |  |  |  |  |  |  |
|  | Length (bp) | 18389 | 18341 | 18372 | 18365 | 18381 | 18349 |
|  | GC content (%) | 36.57 | 36.67 | 36.61 | 36.78 | 36.58 | 36.64 |
|  | Length (%) | 11.68 | 11.73 | 11.67 | 11.62 | 11.68 | 11.67 |

**Additional file 6: Table S5. The basic features of functional regions of cp genomes of six *Viola* species.**

GC, guanine and cytosine; CDS, protein coding sequences.
