# Supplementary material for: An integrated study of Violae Herba (Viola philippica) and five adulterants by morphology, chemical compositions and chloroplast genomes: insights into its certified plant origin
Source: Chin Med. 2022 Mar 3;17:32. doi: 10.1186/s13020-022-00585-9 (PMC8892722; doi:10.1186/s13020-022-00585-9)
Supplement: Supplementary file 7 — Additional file 7: Table S6. Genes contained in each part of the cp genomes of six Viola species. [file 13020_2022_585_MOESM7_ESM.docx]

**Additional file 7: Table S6. Genes contained in each part of the cp genomes of six *Viola* species.**

| **Region** | **Number of CDS** | **Number of tRNA** | **Number of rRNA** | **Total** |
| --- | --- | --- | --- | --- |
| LSC | 59 | 22 | - | 81 |
| SSC | 11 | 1 | - | 12 |
| IRa | 7 | 7 | 4 | 18 |
| IRb | 7 | 7 | 4 | 18 |
| Total | 84 | 37 | 8 | 129 |

CDS gene *rps12* is a trans-spliced gene with its 5’ terminus located in LSC region and the 3’ terminus with a copy located in each of the IR regions.
