# Supplementary material for: An integrated study of Violae Herba (Viola philippica) and five adulterants by morphology, chemical compositions and chloroplast genomes: insights into its certified plant origin
Source: Chin Med. 2022 Mar 3;17:32. doi: 10.1186/s13020-022-00585-9 (PMC8892722; doi:10.1186/s13020-022-00585-9)
Supplement: Supplementary file 8 — Additional file 8: Table S7. The lengths of exons and introns in the cp genomes of six Viola species. [file 13020_2022_585_MOESM8_ESM.docx]

**Additional file 8: Table S7. The lengths of exons and introns in the cp genomes of six *Viola* species.**

| **Gene** | **Location** | **Exon I (bp)** | **Intron I (bp)** | | | | | | | | | **Exon II (bp)** | **Intron II (bp)** | | | | | | **Exon III (bp)** |
| --- | --- | --- | --- | --- | --- | --- | --- | --- | --- | --- | --- | --- | --- | --- | --- | --- | --- | --- | --- |
|  |  |  | ***V.i*** | ***V.b*** | | ***V.j*** | ***V.c*** | | ***V.p*** | | ***V.pr*** |  | ***Vi*** | ***Vb*** | ***Vj*** | ***Vc*** | ***V.p*** | ***V.pr*** |  |
| *atpF* | LSC | 144 | 702 | | 719 | 704 | 704 | | 702 | | 702 | 411 |  |  |  |  |  |  |  |
| *clpP* | LSC | 54 | 843 | | 836 | 844 | 881 | | 843 | | 836 | 306 | 627 | 627 | 627 | 625 | 626 | 622 | 228 |
| *ndhA* | SSC | 551 | 1135 | | 1146 | 1131 | 1159 | | 1134 | | 1135 | 541 |  |  |  |  |  |  |  |
| *ndhB* | IR | 777 | 682 | 686 | | 682 | 686 | | 682 | | 682 | 756 |  |  |  |  |  |  |  |
| *petB* | LSC | 6 | 840 | 831 | | 839 | 857 | | 839 | | 839 | 642 |  |  |  |  |  |  |  |
| *petD* | LSC | 8 | 818 | 812 | | 814 | 813 | | 814 | | 814 | 490 |  |  |  |  |  |  |  |
| *rpl2* | IR | 397^#^ | 671 | 672 | | 671 | 673 | | 671 | | 671 | 434 |  |  |  |  |  |  |  |
| *rpl16* | LSC | 9 | 1160 | 1114 | | 1143 | 1092 | | 1159 | | 1140 | 399 |  |  |  |  |  |  |  |
| *rpoC1* | LSC | 453 | 750 | 749 | | 751 | 762 | | | 750 | 750 | 1626 |  |  |  |  |  |  |  |
| *rps12** | LSC-IR | 114 | - | - | | - | - | | | - | - | 232 | 536 | 536 | 536 | 536 | 536 | 536 | 26 |
| *ycf3* | LSC | 126 | 722 | 721 | | 720 | 722 | | | 720 | 719 | 227 | 710 | 714 | 710 | 711 | 710 | 710 | 154 |
| *trnA-UGC* | IR | 38 | 802 | 802 | | 802 | 802 | | 802 | | 802 | 35 |  |  |  |  |  |  |  |
| *trnG-UCC* | LSC | 23 | 716 | 697 | | 716 | 700 | 716 | | | 716 | 48 |  |  |  |  |  |  |  |
| *trnI-GAU* | IR | 42 | 945 | 945 | | 945 | 945 | | 945 | | 945 | 35 |  |  |  |  |  |  |  |
| *trnL-UAA* | LSC | 37 | 481 | 479 | | 480 | 505 | | 481 | | 481 | 50 |  |  |  |  |  |  |  |
| *trnK-UUU* | LSC | 37 | 2522 | 2523 | | 2523 | 2454 | | 2522 | | 2522 | 35 |  |  |  |  |  |  |  |
| *trnV-UAC* | LSC | 39 | 618 | 618 | | 616 | 623 | | 620 | | 618 | 37 |  |  |  |  |  |  |  |

* trans-spliced gene; # 400 bp in *V. collina*; *V.i: V. inconspicua, V.b: V. betonicifolia, V.j: V. japonica, V.c: Viola collina, V.p: V. philippica, V.pr: V. prionantha.*
