# Supplementary material for: An integrated study of Violae Herba (Viola philippica) and five adulterants by morphology, chemical compositions and chloroplast genomes: insights into its certified plant origin
Source: Chin Med. 2022 Mar 3;17:32. doi: 10.1186/s13020-022-00585-9 (PMC8892722; doi:10.1186/s13020-022-00585-9)
Supplement: Supplementary file 16 — Additional file 16: Table S17. The 12 complete chloroplast genome sequences downloaded from GenBank. [file 13020_2022_585_MOESM16_ESM.docx]

**Additional file 16: Table S17. The 12 complete chloroplast genome sequences downloaded from GenBank.**

| **Species** | **GenBank Accession Number** |
| --- | --- |
| *Viola websteri* Hemsl. | MH229819 |
| *Viola raddeana* Regel | MH229818 |
| *Viola phalacrocarpa* Maxim. | MH229817 |
| *Viola seoulensis* Nakai | KP749924 |
| *Viola mirabilis* L. | MH229816 |
| *Viola selkirkii* Pursh ex Goldie | MW477467 |
| *Viola ulleungdoensis* M. Kim & Jungsim Lee | MW477469 |
| *Viola verecunda* A. Gray | MW586692 |
| *Viola philippica* Cav. | MT796627 |
| *Viola prionantha* Bunge | MT610374 |
| *Viola ulleungdoensis* M. Kim & Jungsim Lee | MK228834 |
| *Passiflora edulis* Sims | KX290855 |
