# Supplementary material for: An integrated study of Violae Herba (Viola philippica) and five adulterants by morphology, chemical compositions and chloroplast genomes: insights into its certified plant origin
Source: Chin Med. 2022 Mar 3;17:32. doi: 10.1186/s13020-022-00585-9 (PMC8892722; doi:10.1186/s13020-022-00585-9)
Supplement: Supplementary file 17 — Additional file 17: Figure S7. NJ trees constructed by 7 variable regions and 2 universal barcodes. [file 13020_2022_585_MOESM17_ESM.pdf]

Additional file 17: Figure S7. NJ trees constructed by 7 variable regions and 2 universal barcodes. Species highlighted in red means that the branch support is below 50%.

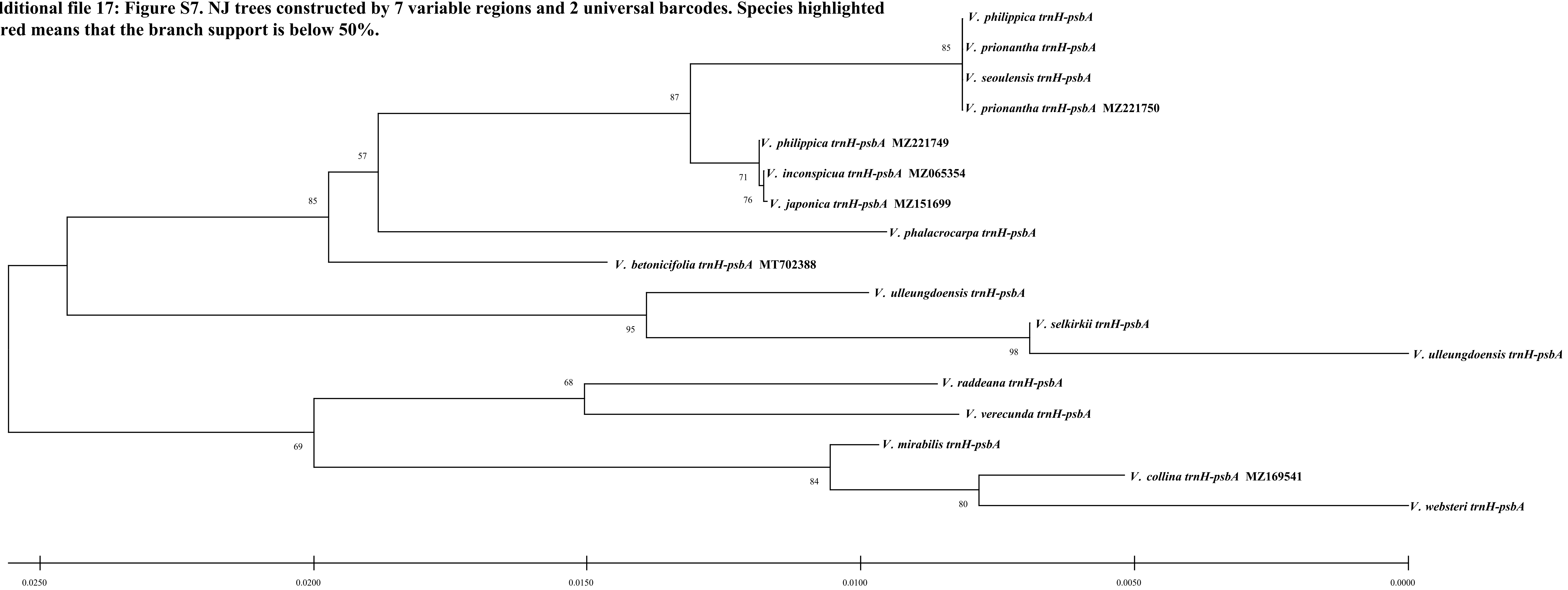

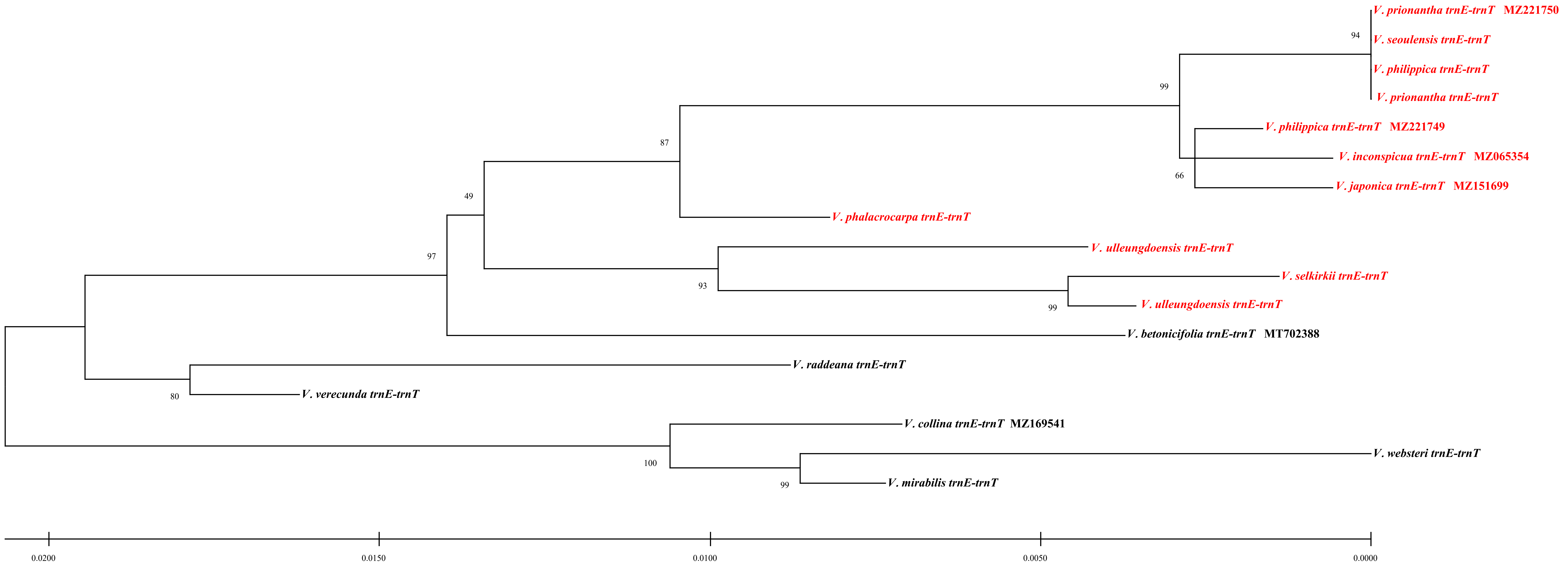

V. prionantha trnE-trnT MZ221750
V. seoulensis trnE-trnT
V. philippica trnE-trnT
V. prionantha trnE-trnT
V. philippica trnE-trnT MZ221749
V. inconspicua trnE-trnT MZ065354
V. japonica trnE-trnT MZ151699
V. phalacrocarpa trnE-trnT
V. ulleungdoensis trnE-trnT
V. selkirkii trnE-trnT
V. ulleungdoensis trnE-trnT
V. betonicifolia trnE-trnT MT702388
V. raddeana trnE-trnT
V. verecunda trnE-trnT
V. collina trnE-trnT MZ169541
V. websteri trnE-trnT
V. mirabilis trnE-trnT
94
99
66
87
49
97
93
99
80
100
99
0.0200
0.0150
0.0100
0.0050
0.0000

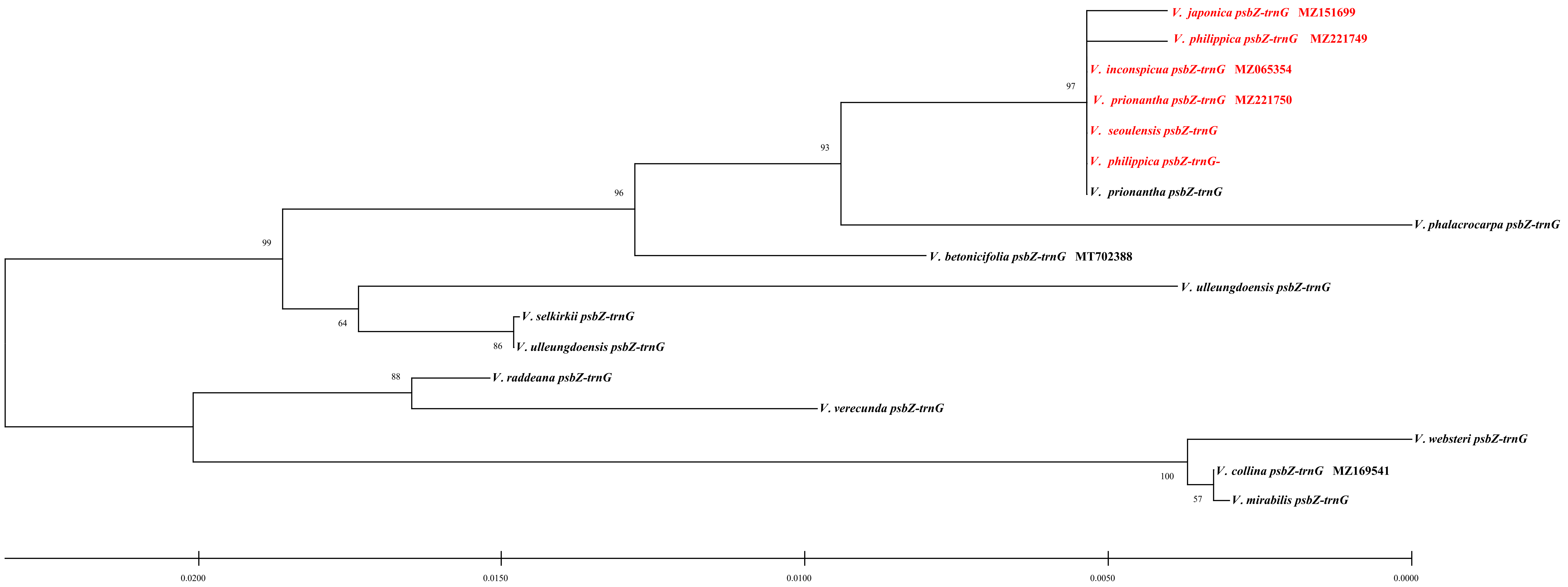

V. japonica psbZ-trnG MZ151699
V. philippica psbZ-trnG MZ221749
V. inconspicua psbZ-trnG MZ065354
V. prionantha psbZ-trnG MZ221750
V. seoulensis psbZ-trnG
V. philippica psbZ-trnG-
V. prionantha psbZ-trnG
V. phalacrocarpa psbZ-trnG
V. betonicifolia psbZ-trnG MT702388
V. ulleungdoensis psbZ-trnG
V. selkirkii psbZ-trnG
V. ulleungdoensis psbZ-trnG
V. raddeana psbZ-trnG
V. verecunda psbZ-trnG
V. websteri psbZ-trnG
V. collina psbZ-trnG MZ169541
V. mirabilis psbZ-trnG
97
93
96
99
64
86
88
100
57
0.0200
0.0150
0.0100
0.0050
0.0000

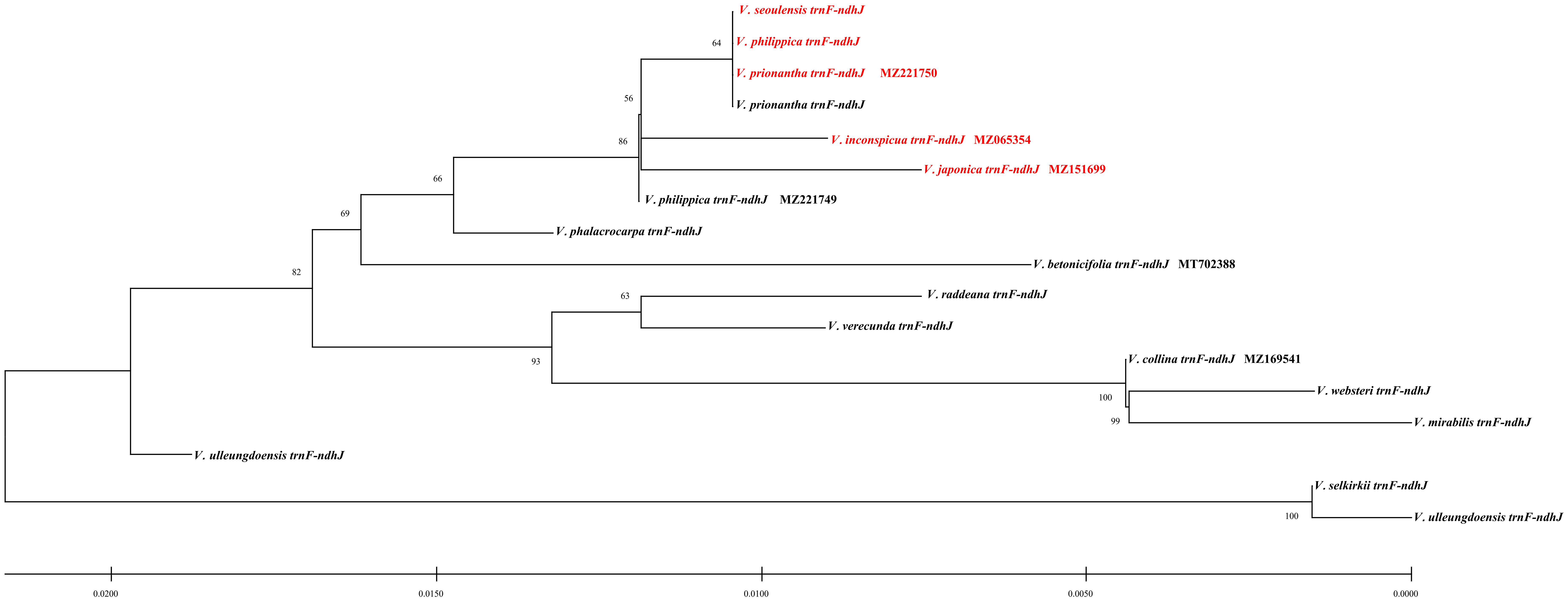
V. seoulensis trnF-ndhJ
64
V. philippica trnF-ndhJ
V. prionantha trnF-ndhJ MZ221750
56
V. prionantha trnF-ndhJ
86
V. inconspicua trnF-ndhJ MZ065354
V. japonica trnF-ndhJ MZ151699
66
V. philippica trnF-ndhJ MZ221749
69
V. phalacrocarpa trnF-ndhJ
82
V. betonicifolia trnF-ndhJ MT702388
63
V. raddeana trnF-ndhJ
V. verecunda trnF-ndhJ
93
V. collina trnF-ndhJ MZ169541
100
V. websteri trnF-ndhJ
99
V. mirabilis trnF-ndhJ
V. ulleungdoensis trnF-ndhJ
V. selkirkii trnF-ndhJ
100
V. ulleungdoensis trnF-ndhJ
0.0200
0.0150
0.0100
0.0050
0.0000

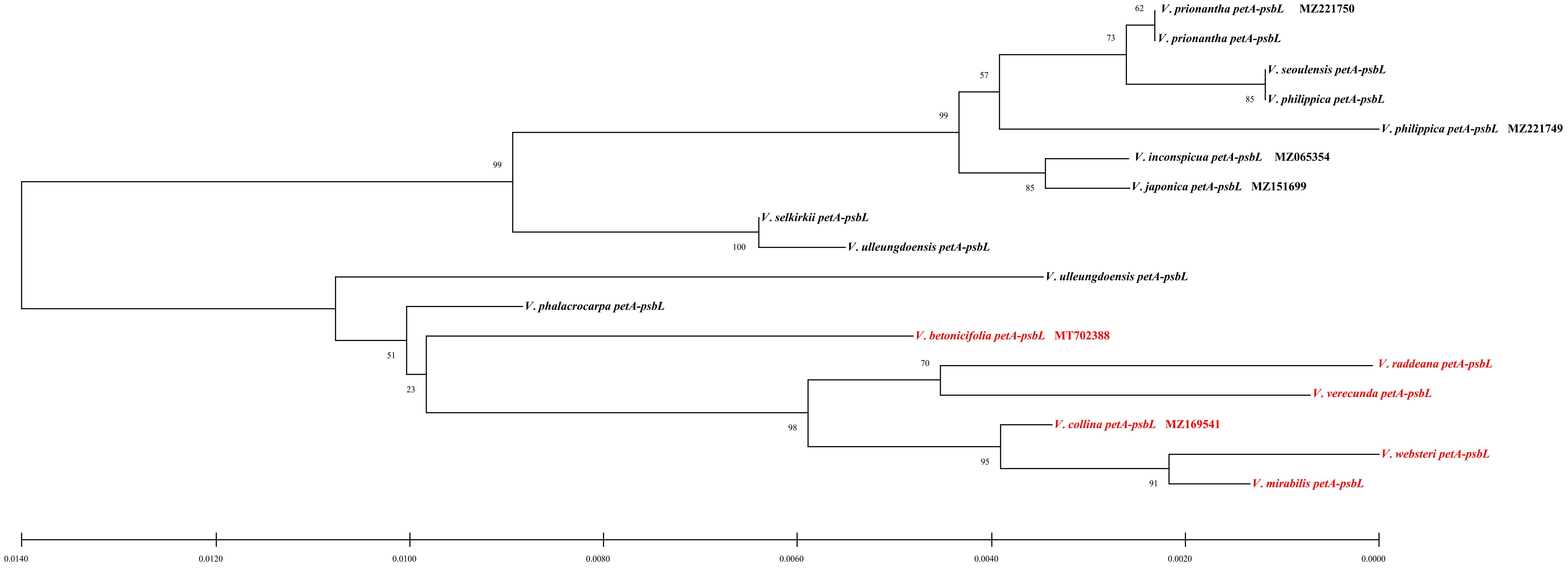

V. prionantha petA-psbL MZ221750
V. prionantha petA-psbL
V. seoulensis petA-psbL
V. philippica petA-psbL
V. philippica petA-psbL MZ221749
V. inconspicua petA-psbL MZ065354
V. japonica petA-psbL MZ151699
V. selkirkii petA-psbL
V. ulleungdoensis petA-psbL
V. ulleungdoensis petA-psbL
V. phalacrocarpa petA-psbL
V. betonicifolia petA-psbL MT702388
V. raddeana petA-psbL
V. verecunda petA-psbL
V. collina petA-psbL MZ169541
V. websteri petA-psbL
V. mirabilis petA-psbL
62
73
57
85
99
99
85
100
51
23
70
98
95
91
0.0140
0.0120
0.0100
0.0080
0.0060
0.0040
0.0020
0.0000

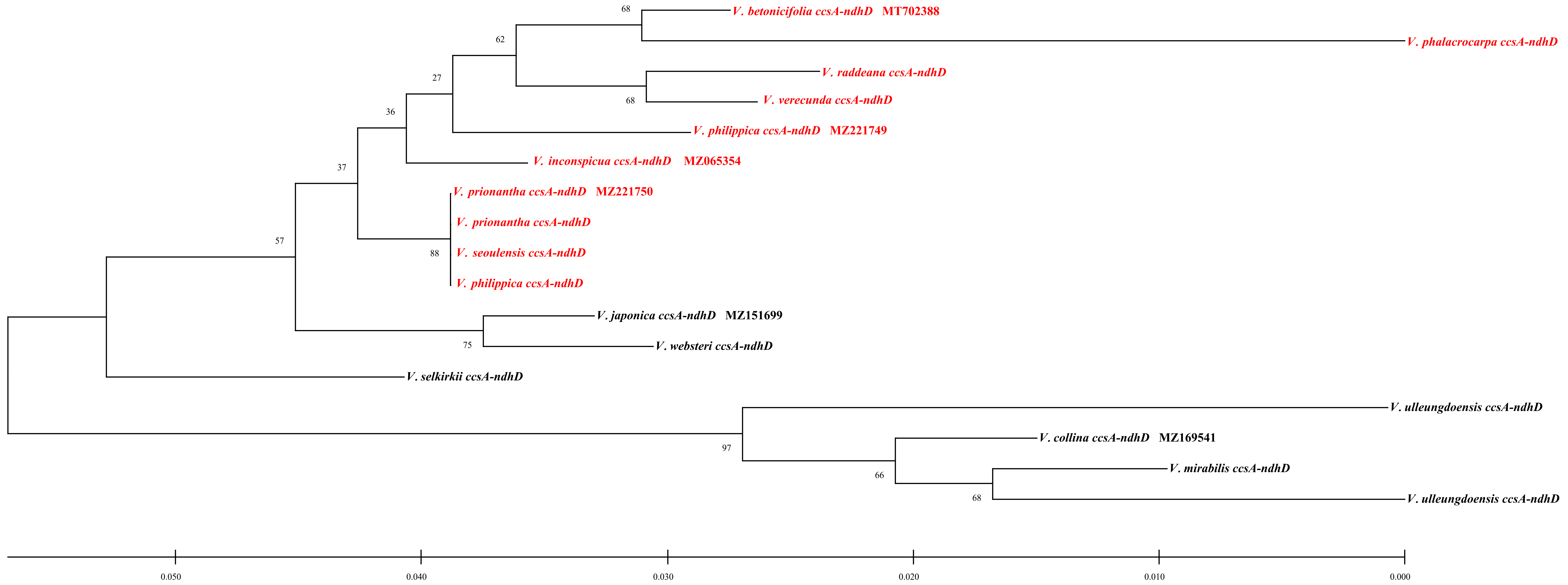
V. betonicifolia ccsA-ndhD MT702388
V. phalacrocarpa ccsA-ndhD
V. raddeana ccsA-ndhD
V. verecunda ccsA-ndhD
V. philippica ccsA-ndhD MZ221749
V. inconspicua ccsA-ndhD MZ065354
V. prionantha ccsA-ndhD MZ221750
V. prionantha ccsA-ndhD
V. seoulensis ccsA-ndhD
V. philippica ccsA-ndhD
V. japonica ccsA-ndhD MZ151699
V. websteri ccsA-ndhD
V. selkirkii ccsA-ndhD
V. ulleungdoensis ccsA-ndhD
V. collina ccsA-ndhD MZ169541
V. mirabilis ccsA-ndhD
V. ulleungdoensis ccsA-ndhD
68
62
27
36
68
37
57
88
75
97
66
68
0.050
0.040
0.030
0.020
0.010
0.000

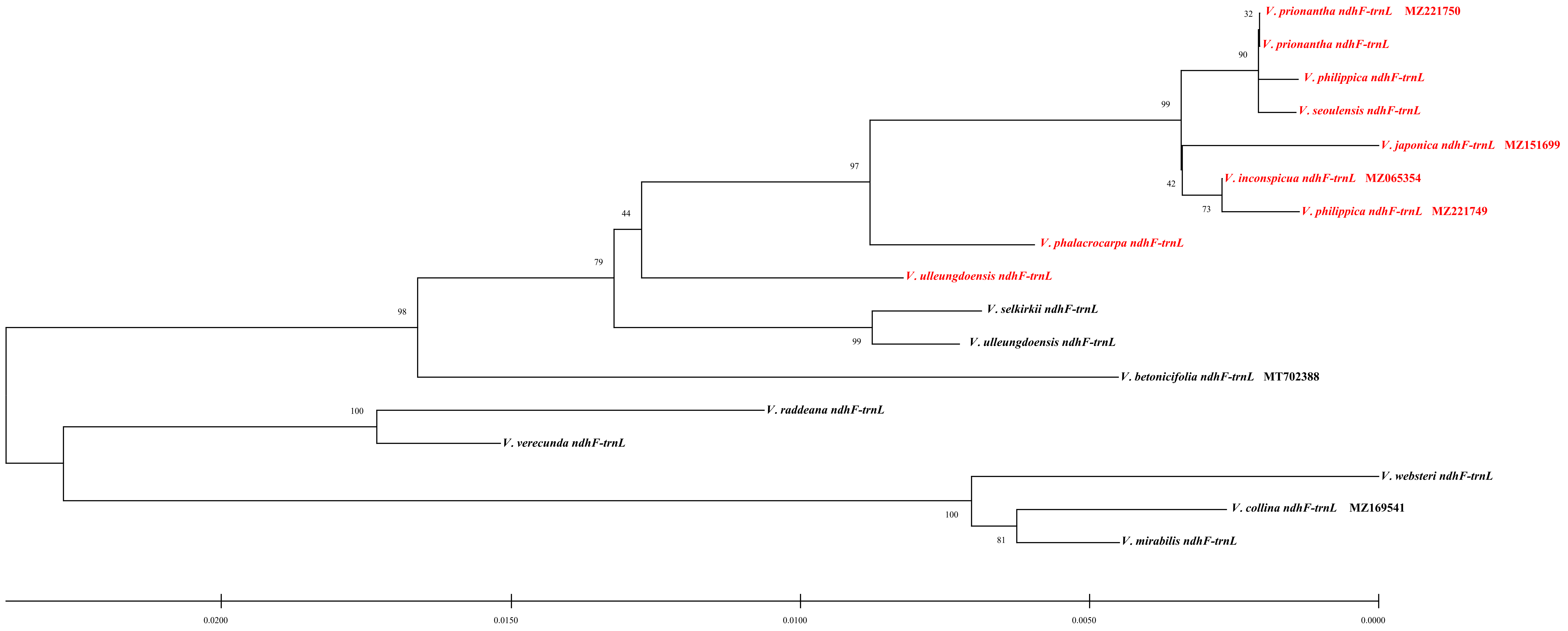

32
V. prionantha ndhF-trnL MZ221750
V. prionantha ndhF-trnL
90
V. philippica ndhF-trnL
99
V. seoulensis ndhF-trnL
V. japonica ndhF-trnL MZ151699
97
42
V. inconspicua ndhF-trnL MZ065354
73
V. philippica ndhF-trnL MZ221749
44
V. phalacrocarpa ndhF-trnL
79
V. ulleungdoensis ndhF-trnL
98
V. selkirkii ndhF-trnL
99
V. ulleungdoensis ndhF-trnL
V. betonicifolia ndhF-trnL MT702388
100
V. raddeana ndhF-trnL
V. verecunda ndhF-trnL
V. websteri ndhF-trnL
100
V. collina ndhF-trnL MZ169541
81
V. mirabilis ndhF-trnL
0.0200
0.0150
0.0100
0.0050
0.0000

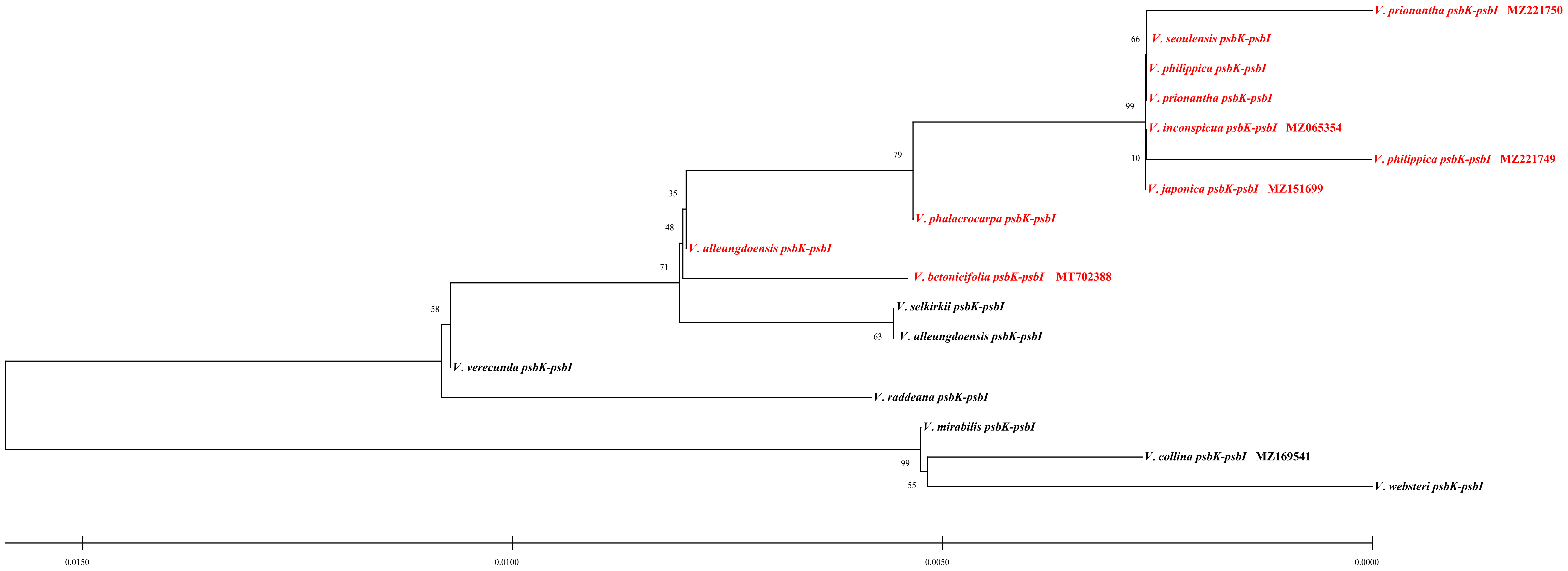
V. prionantha psbK-psbI MZ221750
66
V. seoulensis psbK-psbI
V. philippica psbK-psbI
V. prionantha psbK-psbI
99
V. inconspicua psbK-psbI MZ065354
79
10
V. philippica psbK-psbI MZ221749
V. japonica psbK-psbI MZ151699
35
V. phalacrocarpa psbK-psbI
48
V. ulleungdoensis psbK-psbI
71
V. betonicifolia psbK-psbI MT702388
V. selkirkii psbK-psbI
58
63
V. ulleungdoensis psbK-psbI
V. verecunda psbK-psbI
V. raddeana psbK-psbI
V. mirabilis psbK-psbI
99
V. collina psbK-psbI MZ169541
55
V. websteri psbK-psbI
0.0150
0.0100
0.0050
0.0000

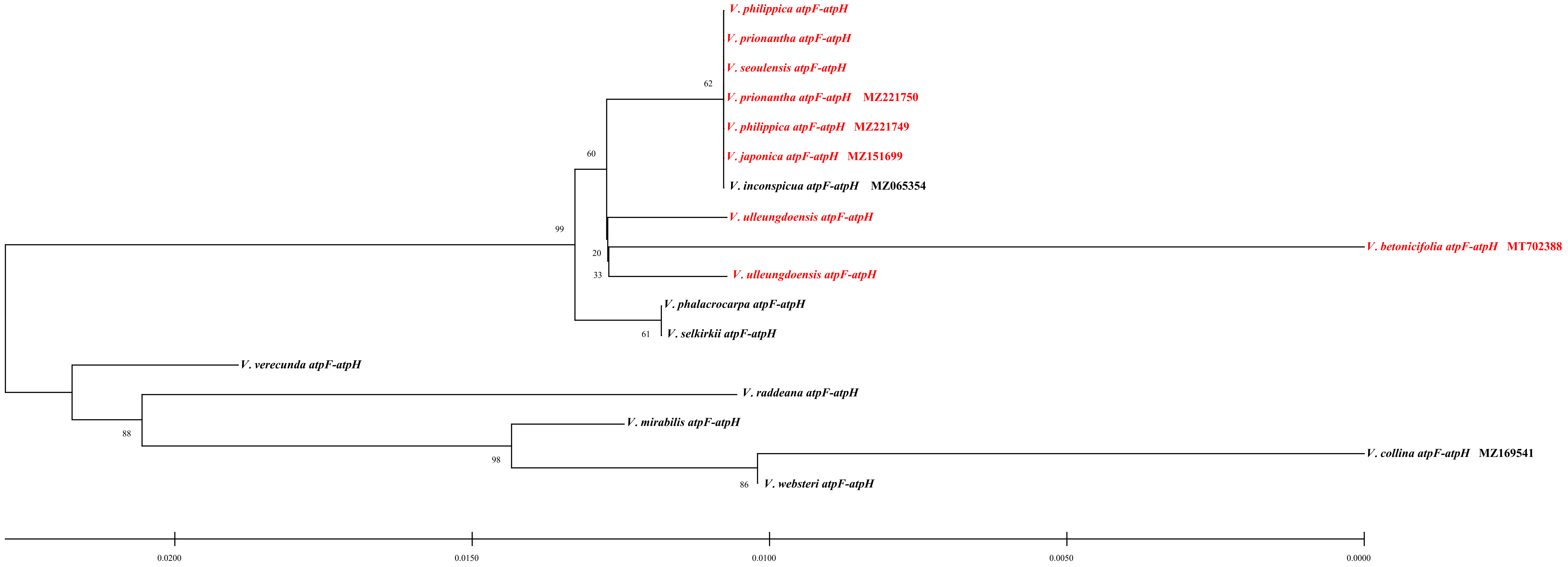

V. philippica atpF-atpH
V. prionantha atpF-atpH
V. seoulensis atpF-atpH
62
V. prionantha atpF-atpH MZ221750
V. philippica atpF-atpH MZ221749
60
V. japonica atpF-atpH MZ151699
V. inconspicua atpF-atpH MZ065354
V. ulleungdoensis atpF-atpH
99
20
V. betonicifolia atpF-atpH MT702388
33
V. ulleungdoensis atpF-atpH
V. phalacrocarpa atpF-atpH
61
V. selkirkii atpF-atpH
V. verecunda atpF-atpH
V. raddeana atpF-atpH
88
V. mirabilis atpF-atpH
98
V. collina atpF-atpH MZ169541
86
V. websteri atpF-atpH
0.0200
0.0150
0.0100
0.0050
0.0000
