# Supplementary material for: An integrated study of Violae Herba (Viola philippica) and five adulterants by morphology, chemical compositions and chloroplast genomes: insights into its certified plant origin
Source: Chin Med. 2022 Mar 3;17:32. doi: 10.1186/s13020-022-00585-9 (PMC8892722; doi:10.1186/s13020-022-00585-9)

Additional file 18: Figure S8. NJ trees constructed by the two-locus universal barcode and three-locus specific barcode. Species highlighted in red means that the branch support is below 50%.

psbK-psbI+atpF-atpH

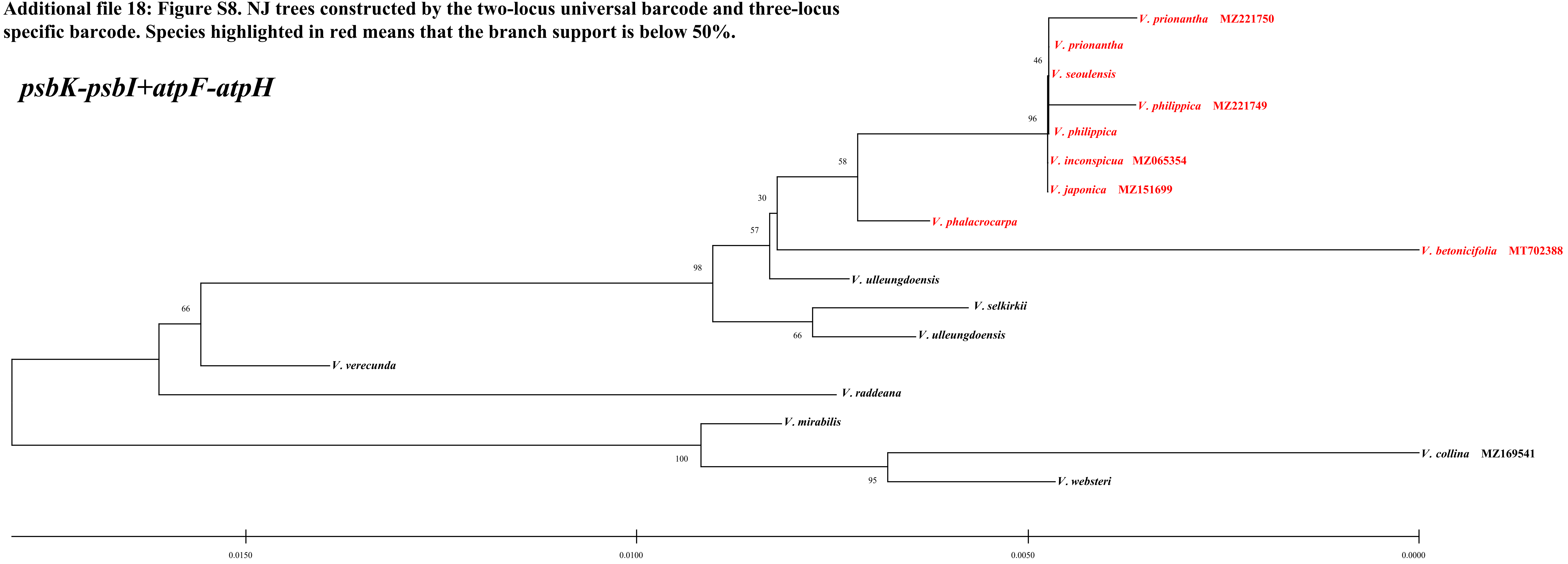

trnH-psbA+trnE-trnT+psbZ-trnG

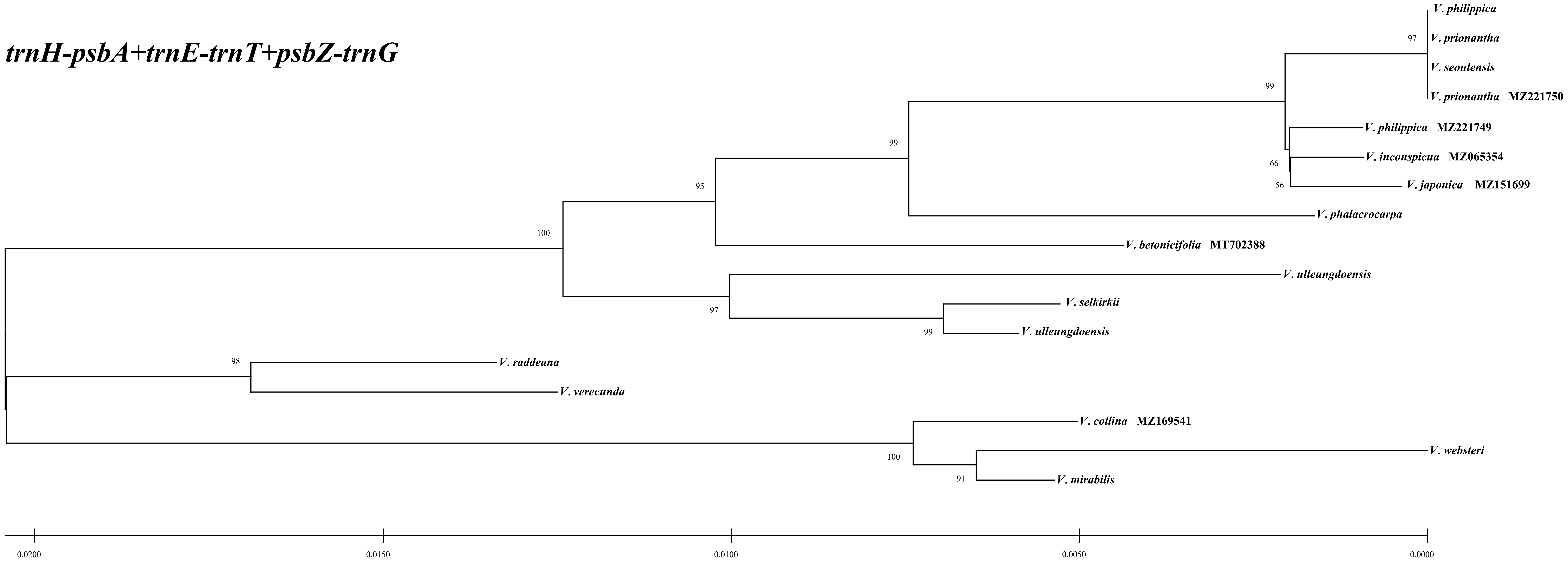

Supplement: Supplementary file 18 — Additional file 18: Figure S8. NJ trees constructed by the two-locus universal barcode and three-locus specific barcode. [file 13020_2022_585_MOESM18_ESM.pdf]
